# Peripheral blood mesenchymal stem cell‐derived exosomes improve renal sympathetic denervation efficacy through β‐catenin‐mediated cardiac reprogramming

**DOI:** 10.1002/ctm2.70475

**Published:** 2025-09-05

**Authors:** Lan Zhao, Chen Li, Zhichuan Huang, Jianshuo Wang, Zhanyu Deng, Yanwen Deng, Pengzhen Wang, Shaoheng Zhang

**Affiliations:** ^1^ Department of Cardiology Guangzhou Red Cross Hospital of Ji‐Nan University Guangzhou China; ^2^ Department of Cardiology Dahua Hospital Shanghai China

**Keywords:** acute myocardial infarction, β‐catenin, cardiac reprogramming, exosome, microRNA

## Abstract

**Background:**

To investigate the role of self‐peripheral blood mesenchymal stem cell (PBMSC)‐derived exosomes (Exos) in enhancing renal sympathetic denervation (RD)‐mediated heart regeneration following myocardial infarction (MI) in a porcine model.

**Methods:**

Pigs (ejection fraction [EF] < 40% post‐MI) were randomised to early sham RD or RD. At 2 weeks post‐MI, autologous PBMSC‐Exos were collected. At 30 days post‐MI, pigs received either PBMSC‐Exos (2 × 10^13^ particles) or phosphate‐buffered saline and were followed until 90 days. Another cohort underwent myocardial biopsy at 14 days post‐MI to assess PBMSC‐Exos effects on ischaemic cardiomyocyte (CM) reprogramming, followed by adeno‐associated viral therapy with miR‐141‐200‐429 sponges or negative control sponges to explore the role of miR‐141‐200‐429 clusters in reprogramming.

**Results:**

Two weeks post‐MI, RD hearts showed increased Exos uptake and inhibited the sympathetic nervous system. By 90 days, the RD+Exos group had 11%–26% higher EF than single‐treatment groups (all *p* < .001), with improved survival and reduced fibrosis. Exos therapy enhanced RD effects by suppressing the renin‒angiotensin‒aldosterone system and transferring the miR‐141‐200‐429 cluster into ischaemic CMs. CMs from RD‐treated hearts cocultured with PBMSC‐Exos^RD^ exhibited a more immature state, promoting reprogramming. β‐Catenin overexpression further enhanced PBMSC‐Exos^RD^ effects, while miR‐141‐200‐429 inhibition blocked RD‐induced CM reprogramming and survival. Ultimately, PBMSC‐Exos^RD^ reduced dickkopf‐1 (Dkk1) expression and activated GSK3β phosphorylation, thereby stimulating the Wnt/β‐catenin pathway.

**Conclusions:**

PBMSC‐Exos^RD^ enhances RD‐mediated cardiac repair through miR‐141‐200‐429 cluster‐dependent activation of the Wnt/β‐catenin pathway, offering a novel therapeutic strategy for MI‐induced heart failure. Our findings unveil a novel therapeutic strategy, highlighting that RD maintains its efficacy and safety when integrated with complementary approaches over extended periods.

**Key points:**

- Myocardial infarction triggers cardiomyocyte depletion and sympathetic overactivation, culminating in irreversible heart failure.
- Renal denervation (RD) attenuates sympathetic signalling, modulating catecholamine‒B‐type natriuretic peptide (BNP) homeostasis.
- We newly demonstrate RD‐enhanced peripheral blood mesenchymal stem cell exosomal secretion enriched with miR‐141‐200‐429 clusters.
- These exosomal miRNAs suppress dickkopf‐1 (Dkk1), activating GSK3β/Wnt/β‐catenin signalling to enhance myocardial survival and regeneration.
- Our findings establish a combined therapeutic paradigm wherein RD maintains durable efficacy and safety alongside complementary interventions for heart failure management.

## Graphical abstract

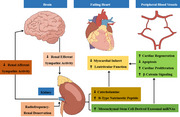

## 1 INTRODUCTION

Percutaneous coronary intervention has the potential to enhance treatment outcomes for myocardial infarction (MI) by enabling timely revascularisation and myocardial salvage; however, it may also lead to cardiomyocyte (CM) loss and activation of the sympathetic nervous system (SNS),^1^ frequently promoting heart failure (HF) progression, with a 5‐year mortality rate of approximately 11%.^2^ Therefore, therapeutic strategies that suppress SNS activation and promote endogenous CM regeneration post‐MI are critically needed.^3^, ^4^

Both cell therapy and renal sympathetic denervation (RD) hold promise for promoting myocardial repair by counteracting functional CM loss and mitigating SNS activation.^5^ RD, a catheter‐based procedure first introduced in 2009 for the treatment of resistant hypertension, involves the ablation of renal sympathetic nerve activity in the kidneys.^6^ Current researches demonstrate that RD can enhance myocardial salvage and improve cardiac function by suppressing SNS activation following MI.^7^ The cardioprotective benefits of RD arise through multiple mechanisms: attenuation of inflammation, inhibition of renin‒angiotensin‒aldosterone system (RAAS), elevation of protective natriuretic peptides, and reduced myocardial fibrosis.^8^ However, to date, there is no evidence supporting RD's ability to modulate cardiac regeneration after MI.

Sustained ischaemia leads to myocardial cell apoptosis and necrosis. Cardiac regeneration research has progressed substantially over 20 years, demonstrating stem/progenitor cells’ ability to produce therapeutic volumes of functional CMs through exogenous administration or endogenous activation.^9^ Mesenchymal stem cells (MSCs) exhibit multilineage potential, giving rise to cardiac, endothelial, and vascular smooth muscle cell types. MSCs have strong paracrine effects, making them promising candidates for endogenous regeneration and repair pathways. Relatively, peripheral blood mesenchymal stem cells (PBMSCs) demonstrate superior advantages over conventional adipose‐ or bone marrow‐derived MSCs, including easier accessibility, reduced invasiveness and enhanced clinical feasibility. Recent trends demonstrate that PBMSCs are a promising source for regenerative medicine favouring ambulatory cell sourcing, as endorsed in the ISCT 2023 Position Statement on translational MSC protocols. We recently revealed that PBMSCs are efficacious regenerative materials, exerting their benefits on ischaemic myocardium via their paracrine effects.^10^ The poor survival of transplanted stem cells in ischaemic myocardium indicates their regenerative benefits stem largely from paracrine mechanisms rather than transdifferentiation.

Beyond cytokines and growth factors, MSCs secrete exosomes (Exos)—nanoscale extracellular vesicles that mediate intercellular communication with local and distant tissues.^11^ Evidence confirms MSC‐secreted Exos and their molecular cargo (e.g., microRNAs) are key regulators of signalling pathways associated with heart repair and regeneration. Furthermore, studies have shown that very few new myocytes are generated in the hearts of adult rodents following ischaemic injury,^12^ and the proliferation of preexisting CMs primarily drives endogenous cardiac regeneration.^13^ Critical knowledge gaps persist regarding PBMSC‐Exos‐mediated regulation of endogenous CM regeneration mechanisms.

Despite promising laboratory results, both Exos therapy and RD face clinical translation barriers including short half‐life and poorly defined targets.^14^, ^15^ Exos treatments aim to restore compromised CMs integrity, whereas RD targets aberrant neural signalling mechanisms that drive HF progression after MI. This research examines innovative strategies to optimise regenerative interventions for post‐infarction cardiac repair. To boost endogenous cardiac repair post‐MI, we preclinically tested combined RD/Exos therapy. First, RD efficacy was initially assessed in porcine MI models. RD primarily modulates early sympathetic overactivation (days 0‒14 post‐MI), leading short‐term improvement of cardiac performance. However, this improvement did not remain significant afterward. Second, building on evidence that exosomal therapy mitigates late remodelling via reduced fibrosis and enhanced CM proliferation,^14^ we propose that early RD+delayed PBMSC‐derived Exos (PBMSC‐Exos) achieves superior regenerative outcomes versus single interventions. Third, using in vitro molecular mechanistic and in vivo animal experiments, we provided the first evidence that after RD, PBMSC‐Exos carry miR‐141‐200‐429 clusters from renal artery (RA) endothelial cells (RAECs) to ischaemic CMs, resulting in CM reprogramming and improving heart function. Targeting the beneficial communication mediated by PBMSC‐Exos or miR‐141‐200‐429 clusters between RAs and damaged hearts may be a novel strategy for improving RD‐initiated cardiac repair.

## 2 METHODS

See Supporting Information Appendix for expanded materials and methods.

### 2.1 Animals and study design

#### 2.1.1 Rat

To investigate the appropriate dose of PBMSC‐Exos used for injection, a preliminary dose ranging experiment was conducted in adult male Sprague‒Dawley rats (200‒250 g). The left anterior descending coronary artery (LAD) was ligated as previously described,^10^ followed by reperfusion after 45 min. Twenty minutes post‐reperfusion, rats received a 100 µL caudal vein injection over 20 s of either phosphate‐buffered saline (PBS) or MI pig‐derived PBMSC‐Exos (5 × 10^12^, 2 × 10^13^ or 5 × 10^13^ in 100 µL PBS). Thirty days later, all the rats underwent echocardiography and then were euthanised for serological and molecular biology testing.

#### 2.1.2 Pig

We induced MI in domestic pigs (40‒50 kg) via ligation of LAD as described previously.^16^ After 45 min of ischaemia, the pigs were subjected to coronary reperfusion, maintained until the endpoint. Pigs with MI exhibiting left ventricular ejection fraction (LVEF) <40% were selected as HF with reduced ejection fraction (HFrEF) models. A total of 40 pigs were randomised into four groups (*n* = 10/group): (1) early RD‐Sham+delayed PBS; (2) early RD‐Sham+delayed Exos; (3) early RD+delayed PBS; and (4) early RD+delayed Exos (Figure S1). As the negative control (NC) of MI surgery, sham‐operated controls (*n* = 5) underwent identical surgical procedures, including thoracotomy and pericardial exposure, but without coronary artery ligation, following the MI model procedures. Subsequently, all sham‐operated pigs received RD treatment. Here, early RD was defined as RD or RD‐Sham administration within 2 h after MI; delayed PBS or Exos injection was performed 30 days after MI.

All experimental protocols and animal experiments were approved by the Institute for Animal Care and Use Committee at Dahua Hospital and the Animal Care and Use Committee of Guangzhou Red Cross Hospital Medical College of Ji‐Nan University, and were conducted in compliance with the NIH Guide for the Care and Use of Laboratory Animals.

### 2.2 Anaesthesia and euthanasia

Pigs received preanaesthetic atropine (30‒50 µg/kg, intramuscular [IM]), sedation with IM ketamine (15‒20 mg/kg) and diazepam (1.5‒2 mg/kg), and maintenance anaesthesia with intravenous (IV) thiopental (1‒2 mg/kg/min).^16^ All animals were euthanised by IV pentobarbital sodium overdose at 90 days post‐MI.

## 3 RESULTS

### 3.1 RD alters the SNS and RAAS in HFrEF pigs

**FIGURE 1:**
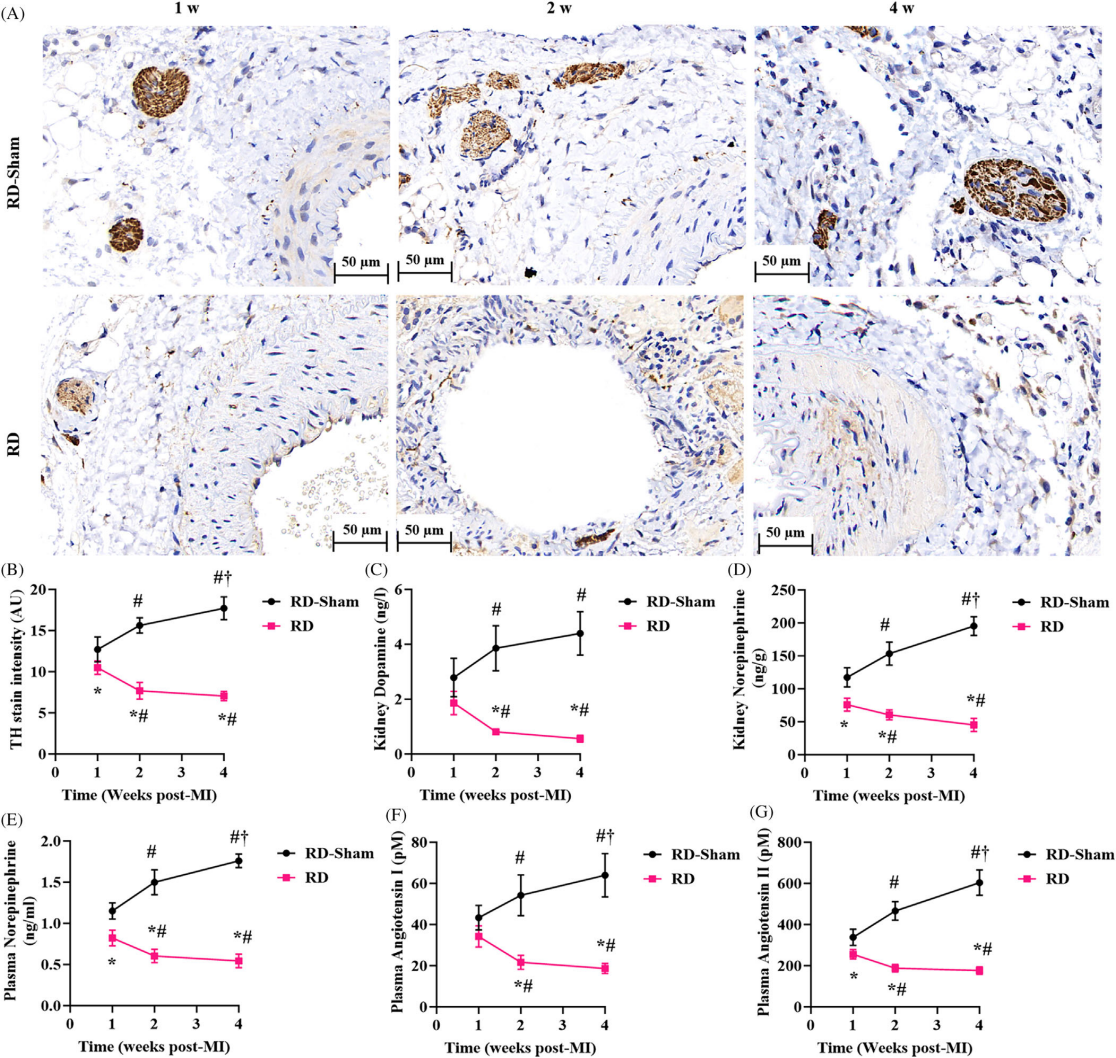
Renal denervation (RD) alters the sympathetic nervous system (SNS) and renin‒angiotensin‒aldosterone system (RAAS). RD induced early‐phase transient suppression of SNS and RAAS activity in myocardial infarction (MI) swine models. (A) Representative images of tyrosine hydroxylase (TH) staining from serial RA samples from RD‐Sham or RD pigs obtained 1, 2 and 4 weeks after MI. Scale bar: 50 µm. (B) TH staining intensity. (C) Dopamine and (D) norepinephrine (NE) concentrations per gram of kidney cortex tissue. Quantitative analysis of plasma NE (E), angiotensin I (F) and angiotensin II (G) 1, 2 and 4 weeks after MI in RD‐Sham or RD pigs. All data, presented as means ± standard deviations, were analysed using one‐way analysis of variance. *p *< .05: ^*^versus RD‐Sham at each timepoint, ^#^ versus 1 week after MI, ^†^versus 2 weeks after MI (*n* = 3 per group).

We first examined temporal changes in plasma catecholamine and angiotensin levels following RD in HFrEF pigs. Tyrosine hydroxylase (TH) immunohistochemistry of renal arterial nerves revealed increased neural integrity after RD‐Sham, but significant denervation after active RD (Figure 1A). In particular, TH staining intensity was the lowest in RD pigs after 2 weeks of MI (Figure 1B); this observation was corroborated by the maximal reduction in renal dopamine and norepinephrine (NE) levels at this time point, which serves as a quantitative measure of sympathetic nerve function (Figure 1C,D). The most significant reduction in circulating NE levels was also noted 2 weeks after RD (Figure 1E). Next, we assessed RAAS activity based on plasma angiotensin I and II levels and noted a significant reduction in their levels in RD pigs, with the lowest levels noted 2 weeks after MI (Figure 1F,G).

Serial assessments revealed no significant alterations in SNS activity or RAAS markers beyond 2 weeks post‐RD, with effects remaining non‐significant at 4 weeks in HFrEF pigs.

### 3.2 RD enhances PBMSC‐derived exosomal release with cardioprotective microRNA cargo in a post‐MI HFrEF porcine model

**FIGURE 2:**
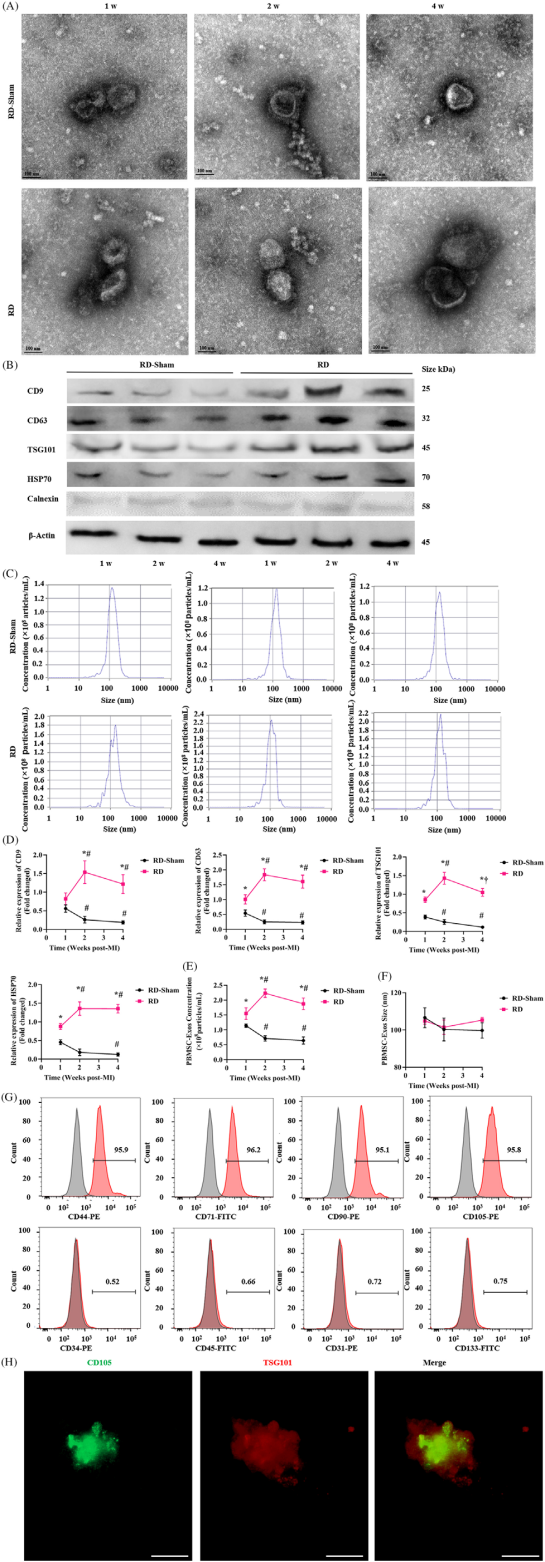
Peripheral blood mesenchymal stem cell (PBMSC)‐exosomes (Exos) viable content after renal denervation (RD) in heart failure with reduced ejection fraction (HFrEF) pigs. RD augments the secretion of PBMSC‐originated Exos in porcine models of HFrEF. (A) Serial representative transmission electron microscopy (TEM) images of PBMSC‐Exos from RD or RD‐Sham pigs 1, 2 and 4 weeks after myocardial infarction (MI). Scale bar: 100 nm. (B) Western blotting for Exos markers CD9, CD63, TSG101 and HSP70, and negative marker Calnexin in PBMSC‐Exos from RD or RD‐Sham pigs 1, 2 and 4 weeks after MI. (C) Size distribution of PBMSC‐Exos analysed by nanoparticle tracking analysis (NTA) 2 weeks post‐MI. (D) Relative expression of Exos marker proteins (CD9, CD63, TSG101, HSP70) in PBMSC‐Exos derived from equal volumes of culture medium from RD or RD‐Sham pigs at 1, 2 and 4 weeks post‐MI, assessed by Western blot. (E) Quantification of PBMSC‐Exos particle concentration in culture medium from RD or RD‐Sham pigs via NTA at 1, 2 and 4 weeks post‐MI. (F) RD or RD‐Sham treatment did not significantly affect the particle size of PBMSC‐Exos from RD or RD‐Sham pigs 1, 2 and 4 weeks after MI. Data represent mean ± standard deviation (SD) (*n* = 3 per group). Statistical significance was determined by one‐way analysis of variance (ANOVA): ^*^ *p* < .05 versus RD‐Sham at the same timepoint; ^#^ *p* < .05 versus RD‐Sham 1 week post‐MI; ^†^ *p* < .05 versus RD‐Sham 2 weeks post‐MI. (G) Flow cytometry analysis of PBMSC‐positive surface markers for the mesenchymal stem cell (MSC) markers CD44, CD71, CD90 and CD105 and negative markers for the haematopoietic stem cell (HSC) markers CD34 and CD45 and EPC markers CD31 and CD133. (H) PBMSC‐Exos were examined via immunofluorescence for the expression of the MSC marker CD105 (green) and Exos markers TSG101 (red). Scale bar: 10 µm.

Since MSC cardioprotection is contact independent,^17^ we proposed Exos deliver bioactive cargo mediating RD's remote effects. To assess the impact of RD on PBMSC‐Exos, Exos were isolated via ultracentrifugation from RD and RD‐Sham pigs at 1, 2, and 4 weeks post‐MI, followed by characterisation using transmission electron microscopy (Figure 2A). Then, Western blotting confirmed exosomal markers (CD9, CD63, TSG101 and HSP70) and absence of Calnexin in PBMSC‐Exos from RD/RD‐Sham pigs at 1, 2, and 4 weeks post‐MI (Figure 2B). CD9 and CD63 were selected as canonical exosomal markers based on MISEV2018 guidelines,^18^ with TSG101 immunoblotting confirming the endosomal origin.^19^ These tetraspanins were prioritised given their established role in CM‒Exos interactions. Statistical analysis revealed spatiotemporal changes in the expression levels of these four marker proteins in RD pigs. Expression peaked at 2 weeks post‐MI, significantly exceeding levels in RD‐Sham pigs at that time. However, by 4 weeks post‐infarction, expression in the RD group had significantly decreased. In the RD‐Sham pigs, expression of these proteins was significantly reduced by the 2‐week time point (Figure 2D). These results were consistent with our nanoparticle tracking analysis (NTA) results: RD increased the expression of these proteins, peaking 2 weeks after MI; in contrast, RD‐Sham gradually reduced their expression, with the greatest decrease occurring 2 weeks after MI (Figure 2C,E). Particle size distributions quantified by NanoSight showed consistent 100 nm mean diameters (unimodal) in both RD and RD‐Sham PBMSC‐Exos across timepoints, without significant intergroup variation (Figure 2C,F)—confirming typical exosomal size parameters.^20^ To confirm that Exos were derived from PBMSCs, we first validate the identity of PBMSCs using cell surface marker profiling by fluorescence‐activated cell sorting (FACS). FACS analysis confirmed that PBMSCs isolated at 2 weeks post‐MI expressed ≥95% mesenchymal markers (CD44, CD71, CD90 and CD105), while showing ≤2% expression of haematopoietic stem cell (CD34 and CD45) and endothelial progenitor cell (EPC) markers (CD31 and CD133) (Figure 2G). Next, we performed co‐immunostaining of the MSC marker protein CD105 and the Exos marker protein TSG101 to confirm that the Exos were derived from PBMSCs. Overexpression of MSC‐specific molecule CD105 in Exos (Figure 2H) confirmed their derivation from PBMSCs.

Taken together, these results indicated that RD increases Exos release that originated from PBMSCs in a time‐dependent manner, with the greatest increase occurring 2 weeks after MI. As such, we selected autologous PBMSC‐Exos from the RD and RD‐Sham group (denoted as PBMSC‐Exos^RD‐Sham^ or PBMSC‐Exos^RD^, respectively) at 2 weeks after MI as the Exos source to investigate the additive effects of delayed exosomal therapy (i.e., intravenous injection of PBMSC‐Exos 30 days after MI) with RD‐induced myocardial repair in our HFrEF pigs.

**FIGURE 3:**
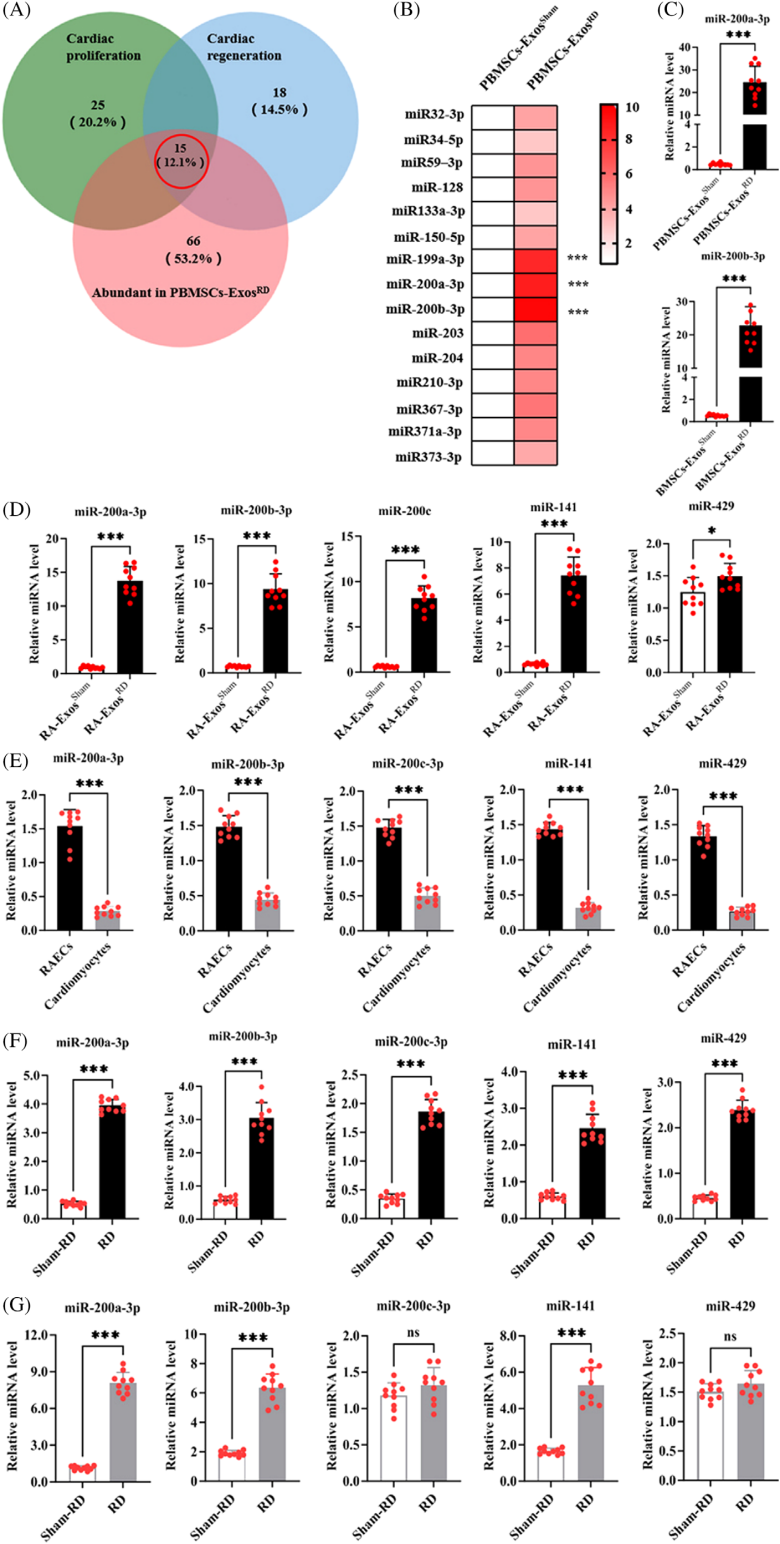
miR‐141‐200‐429 clusters are upregulated in renal denervation (RD) renal artery endothelial cells (RAECs) and transferred to cardiomyocytes (CMs) by peripheral blood mesenchymal stem cell (PBMSC)‐exosomes (Exos). RD enhances the release of PBMSC‐Exos from RAECs, delivering cardioprotective microRNA cargo to infarcted hearts. (A) Venn diagram identifying 15 miRNAs associated with cardiac proliferation and regeneration enriched in the heart. (B) Heatmap generated using correlation distance based on average miRNA expression detected by reverse transcription quantitative real‐time polymerase chain reaction (RT‐qPCR). RT‐qPCR revealed significant miR‐200a‐3p and miR‐200b‐3p elevation in PBMSC‐Exos^RD^ compared with PBMSC‐Exos^RD‐Sham^ from equal amounts of PBMSCs. Relative abundance of each miRNA is indicated by a gradient of colour from red (highest abundance) to white (lowest abundance). ^***^ *p *< .001, multiple *t* tests; *n* = 10. (C) RT‐qPCR revealing significant elevation of miR‐200a‐3p and miR‐200b‐3p expression in PBMSC‐Exos^RD^ compared with equivalent PBMSC‐Exos^RD‐Sham^. (D) Significant elevation of 141‐200‐429 cluster expression in RA‐Exos^RD^ compared with equivalent RA‐Exos^RD‐Sham^. (E) Increased miR‐141‐200‐429 cluster expression in RAECs versus CMs after RD treatment. Relative expression of miR‐141‐200‐429 cluster in renal arteries (RAs) (F) and hearts (G) from RD and RD‐Sham pigs. (C‒G) Data represent mean ± standard deviation (SD) (*n* = 10 per group). Statistical significance was determined by unpaired *t* tests: ^*^ *p *< .05, ^***^ *p *< .001, compared with RD‐Sham; *n* = 10.

Given that numerous miRNAs are known to stimulate cardiac repair by promoting CM dedifferentiation and proliferation after MI,^21^ we conducted small RNA sequencing (RNA‐seq) to characterise miRNAs present in PBMSC‐Exos^RD^. First, to identify miRNAs within PBMSC‐Exos^RD^ that potentially enhance cardiac proliferation and regeneration, we compared 66 miRNAs enriched in PBMSCs with datasets of miRNAs implicated in cardiac proliferation^4^, ^21^ and regeneration.^22^ As illustrated in the Venn diagram (Figure 3A), we identified 15 common miRNAs. Next, we analysed the expression of these 15 miRNAs in PBMSC‐Exos^RD^ versus PBMSC‐Exos^RD‐Sham^ and noted that PBMSC‐Exos^RD^ significantly upregulated the expression of miR‐199a‐3p (7.73‐fold), miR‐200a‐3p (8.03‐fold) and miR‐200b‐3p (9.01‐fold) compared to PBMSC‐Exos^RD‐Sham^ (Figure 3B). Third, to determine whether the observed increase in miRNA expression was due to a higher number of PBMSC‐Exos^RD^ or an increase in their miRNA content, we quantified miRNA expression per equal number of PBMSC‐Exos^RD^ using real‐time quantitative polymerase chain reaction (RT‐qPCR). Significantly elevated expression of miR‐200a‐3p and miR‐200b‐3p was observed in PBMSC‐Exos^RD^ compared to an equivalent amount of PBMSC‐Exos^RD‐Sham^ (Figure 3C). Both miRNAs are members of the kidney‐enriched miR‐141‐200‐429 cluster,^23^ which includes miR141and miR429.^24^ To investigate whether RD‐treated RAs release PBMSC‐Exos^RD^ with elevated miR‐200 content, we measured the expression of the miR‐141‐200‐429 expression in Exos derived from RA (RA‐Exos). Compared to RA‐Exos^RD‐Sham^, RA‐Exos^RD^ exhibited significantly higher levels of all miR‐141‐200‐429 cluster members (Figure 3D). Finally, miR‐141‐200‐429 cluster expression were compared between RAs and hearts in RD‐treated pigs. All five miRNAs analysed (miR‐200a‐3p, miR‐200b‐3p, miR‐200c‐3p, miR‐141 and miR‐429) showed significantly higher expression in RAECs compared to CMs (Figure 3E). Following RD, expression of these miRNAs was significantly increased specifically in RAECs, with miR‐200a‐3p (6.52‐fold) and miR‐200b‐3p (4.23‐fold), exhibiting the strongest upregulation (Figure 3F). Conversely, in CMs, RD significantly upregulated only miR‐141, miR‐200a‐3p and miR‐200b‐3p (Figure 3G). miR‐200b‐3p‐encapsulated MSC‐derived exosomes (MSC‐Exos) have been shown to protect against MI‐induced CM apoptosis and inflammation.^25^ These findings collectively suggest that RD stimulates the release of PBMSC‐Exos from RAECs, delivering cardioprotective microRNA cargo to infarcted hearts.

### 3.3 RD improves short‐term cardiac performance and delayed exosomal therapy affords long‐term benefit

**FIGURE 4:**
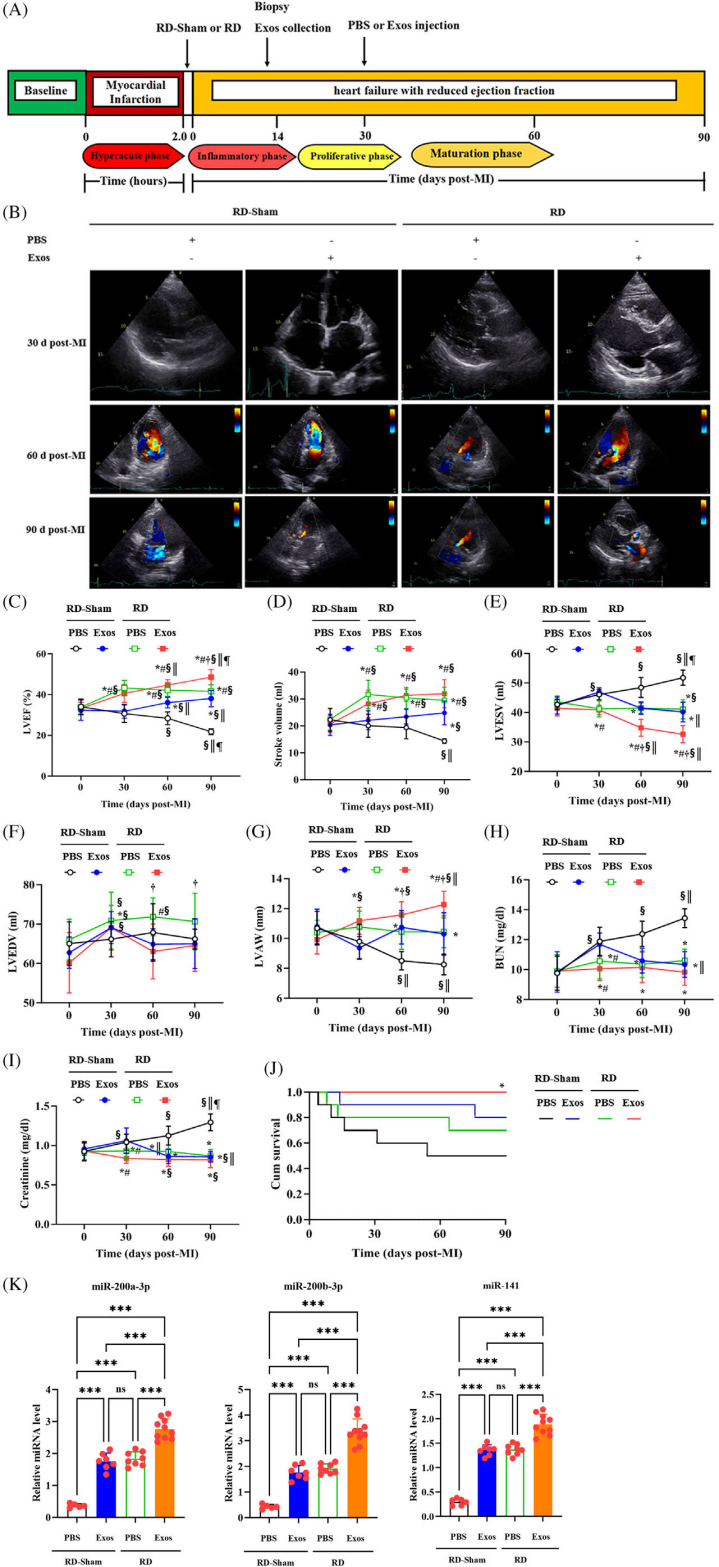
Delayed exosomal therapy promotes improved outcome in the renal denervation (RD)‐treated pigs. Myocardial exosomal miR‐141‐200 cluster mediates the therapeutic effects of both RD and exosomes (Exos) interventions on cardiac functional restoration. (A) Ischaemic heart failure with reduced ejection fraction (HFrEF) protocol. (B) Representative echocardiography images of hearts from pigs receiving RD‐Sham or RD with or without delayed mesenchymal stem cell (MSC)‐derived Exos transplantation 30, 60 and 90 days after myocardial infarction (MI). (C‒G) Echocardiography for left ventricular ejection fraction (LVEF) (C), stroke volume (D), left ventricular end‐systolic volume (LVESV) (E), left ventricular end‐systolic volume (LVEDV) (F) and left ventricular anterior wall (LVAW) (G) 0, 30, 60 and 90 days after MI. (H and I) Circulating blood urea nitrogen (BUN) (H) and creatinine (I) levels on 0 days, 30 days after MI, 60 days after MI and 90 days after MI. All data, presented as means ± standard deviations, were analysed using one‐way analysis of variance (ANOVA). *p *< .05: ^*^versus RD‐Sham+PBS, ^#^versus RD‐Sham+Exos, ^†^versus RD+PBS, ^§^versus 0 days after MI, ^║^versus 30 days after MI, ^¶^versus 60 days post‐MI; ns, non‐significant. (0 days after MI, *n* = 10 pigs in each group; 30 days after MI, *n* = 7 for RD‐Sham+PBS, *n* = 8 for RD‐Sham+Exos, *n* = 9 for RD‐Sham+PBS and *n* = 10 for RD+Exos; 60 days after MI, *n* = 5 for RD‐Sham+PBS, *n* = 8 for RD‐Sham+Exos, *n* = 9 for RD‐Sham+PBS and *n* = 10 for RD+Exos; 90 days after MI, *n* = 5 for RD‐Sham+PBS, *n* = 7 for RD‐Sham+Exos, *n* = 8 for RD‐Sham+PBS and *n* = 10 for RD+Exos). (J) Kaplan‒Meier survival curves to compare mortality between the four pig groups. Significance was determined by log‐rank (Mantel‒Cox) test (*n* = 10 per group). (K) Relative expression of miR‐141‐200‐429 cluster in hearts from pigs receiving RD‐Sham or RD with or without delayed MSC‐derived Exos transplantation 90 days after MI. All data, presented as means ± standard deviations, were analysed using one‐way ANOVA. ^***^ *p *< .001; ns, non‐significant (*n* = 5 for RD‐Sham+PBS, *n* = 7 for RD‐Sham+Exos, *n* = 8 for RD‐Sham+PBS and *n* = 10 for RD+Exos). PBS, phosphate‐buffered saline.

Confirming our earlier report, PBMSCs (5 × 10^6^ cells) improved cardiac function.^10^ By applying this cell count, we had obtained 2 × 10^13^ PBMSC‐Exos from MI pigs. Next, we further tested this dosage in MI rats. PBMSC‐Exos were injected into caudal vein of MI rats at dosage of 5 × 10^12^, 2 × 10^13^ or 5 × 10^13^ in 100 µL PBS. At doses of 2 × 10^13^ and 5 × 10^13^ particles/kg (but not 5 × 10^12^), PBMSC‐Exos improved cardiac function (LVEF), upregulated β‐catenin and Oct4 expression, and reduced CMs injury (plasmas‐cTnT) (Figure S2). Given the equivalent efficacy at both higher doses, we proceeded with the 2 × 10^13^ dose in subsequent pig experiments to assess cardiac repair. Figure 4A presents a schematic timeline integrating RD/Exos intervention windows with pathophysiological milestones. Based on the above results, we harvested PBMSC‐Exos at day 14 post‐MI, and we aimed to capture Exos with maximal reparative cargo before potential functional exhaustion. We performed RD immediately after MI for modulating early sympathetic overactivation (days 0‒14 post‐MI), which exacerbates inflammation. Then, Exos therapy focuses on the transition period between proliferation and scar formation (day 30 post‐MI).^26^

In this study, MI was induced in 82 out of 95 experimental pigs by ligating LAD. From these, 40 pigs with LVEF <40% were selected and randomly assigned to receive early (within 2 h after MI) RD‐Sham or RD followed by delayed (30 days after MI) PBS or Exos injection, with 10 pigs per group. These groups were labelled as RD‐Sham+PBS, RD‐Sham+PBMSC‐Exos^RD‐Sham^ (denoted as RD‐Sham+Exos), RD+PBS and RD+PBMSC‐Exos^RD^ (denoted as RD+Exos). No deaths occurred in this cohort of pigs during any treatment. However, 10 pigs died during follow‐up. Finally, 30 pigs survived to undergo serial functional studies 90 days after MI (Figure S1).

Functional data, measured through echocardiography, demonstrated that early RD administration after MI significantly improved LVEF 30 days after MI compared with early RD‐Sham; however, this improvement did not remain significant afterward. Although delayed Exos injection significantly improved LVEF in the RD‐Sham+Exos group by 60 days post‐MI, the therapeutic effect failed to persist until 90 days post‐MI. However, sequential Exos therapy maintained these therapeutic benefits throughout the study period (Figure 4B,C). At 90 days post‐MI, the RD+Exos group exhibited 11%‒26% greater preservation of LVEF compared to other three groups, with all intergroup differences achieving statistical significance (*p* < .001; Figure 4C). Similarly, at 90 days post‐MI, RD+Exos treatment increased left ventricular stroke volume versus other groups (Figure 4D), attributable to reduced LV end‐diastolic (Figure 4E) and end‐systolic volumes (Figure 4F) and restored LV anterior wall thickness (Figure 4G). These improvements were absent in RD+PBS versus RD‐Sham+PBS comparisons (Figure 4E–G). These cardiac structure and function indices showed no significant differences when comparing the RD‐Sham+Exos group with the RD+PBS group at 90 days after MI. Critically, no deaths occurred during RD or Exos administration procedures. All mortalities occurred during follow‐up, with five deaths in the RD‐Sham+PBS group, three in RD‐Sham+Exos and two in RD‐Sham+PBS. Strikingly, all pigs receiving early RD plus delayed Exos survived the 90‐day post‐MI period, whereas 50% (5/10) of RD‐Sham+PBS controls died within 90 days post‐MI (Figure 4J). Beyond functional parallels, myocardial miR‐200a‐3p, miR‐200b‐3p and miR‐141 levels were markedly elevated following RD or Exos monotherapy. Maximal induction occurred in RD+Exos pigs under combination therapy at 90 days post‐MI (Figure 4K). Critically, miR‐200c‐3p and miR‐429 exhibited no discernible expression alterations across groups (data not shown). These findings aligned with our previous observations in CM‐Exos derived from MI models, strongly suggesting that the myocardial exosomal miR‐141‐200 cluster mediates the therapeutic effects of both RD and Exos interventions on cardiac functional restoration.

Renal function was evaluated by measuring plasma levels of blood urea nitrogen (BUN) and creatinine at 0, 30, 60 and 90 days post‐MI (Figure 4H,I). As HFrEF progressed in the RD‐Sham+PBS group, both BUN and creatinine levels showed progressive elevations. However, RD treatment alone did not adversely affect renal function. Notably, plasma levels of BUN and creatinine in the RD+Exos group were significantly lower than those in the RD‐Sham+PBS group at 30, 60 and 90 days post‐MI. This suggests that the combination of RD and Exos may provide additional reduction in plasma BUN and creatinine levels compared to the RD‐Sham+PBS condition.

Furthermore, the dynamic haemodynamics were monitored in all animals (Table 1). Compared with the baseline level, MI‐induced HF caused a significant increase in heart rate, which persisted in the RD‐Sham+PBS group, but were restored to the baseline levels in the RD‐Sham+Exos group and all RD groups at 90‐day post‐MI. Mean arterial blood pressure showed a gradual decline in the RD‐Sham+PBS group over time, in contrast to the stable levels observed in the other three groups. We also observed that RD did not significantly affect cardiac function, blood pressure and heart rate in pigs that received sham surgery (Table S3). Following RD, no significant alterations were detected in plasma NE or RAAS components (angiotensin I and II), with no biochemical evidence of HF (B‐type natriuretic peptide [BNP] < 100 pg/mL) or myocardial necrosis (cardiac troponin I [cTnI] < .01 ng/mL) (Table S3). All these data suggested that RD‐mediated cardiac repair specifically targets MI‐induced sympathetic hyperactivation rather than non‐ischaemic surgical effects.

**TABLE 1:** Heart rate, mean arterial blood pressure (MABP), B‐type natriuretic peptide (BNP) and hs‐cTnT (means ± standard deviation [SD]).

Time	*n*	Heart rate (beats/min)	MABP (mmHg)	Plasma BNP (pg/mL)	hs‐cTnT (ng/L)
Baseline					
RD‐Sham+PBS	10	85 ± 8	87 ± 4	94.6 ± 19.7	6.4 ± 1.9
RD‐Sham+Exos	10	80 ± 6	85 ± 6	88.6 ± 17.0	7.4 ± 1.3
RD+PBS	10	81 ± 6	85 ± 6	87.0 ± 15.9	7.3 ± 1.4
RD+Exos	10	83 ± 7	84 ± 4	83.1 ± 15.1	7.2 ± 1.3
On the day of MI					
RD‐Sham+PBS	10	105 ± 10	83 ± 5	432.5 ± 84.0^§^	1191.6 ± 209.7^§^
RD‐Sham+Exos	10	108 ± 10	86 ± 6	425.7 ± 83.4^§^	1154.5 ± 248.7^§^
RD+PBS	10	100 ± 9	83 ± 5	436.7 ± 78.2^§^	1150.7 ± 165.6^§^
RD+Exos	10	99 ± 12	81 ± 5	433.4 ± 83.2^§^	1119.6 ± 159.8^§^
30 days post‐MI					
RD‐Sham+PBS	7	115 ± 11^§║^	83 ± 6	528.9 ± 83.0^§^	42.1 ± 12.3^§║^
RD‐Sham+Exos	8	102 ± 13^*§^	85 ± 6	779.4 ± 94.4^*§║^	14.7 ± 4.4^*║^
RD+PBS	9	93 ± 9^*#§^	85 ± 6	687.6 ± 118.5^§║^	17.0 ± 5.9^*║^
RD+Exos	10	95 ± 9^*§^	80 ± 7	829.1 ± 128.8^*§║^	11.3 ± 4.9^*║^
60 days post‐MI					
RD‐Sham+PBS	5	116 ± 8^§║^	81 ± 6^§^	577.8 ± 79.0^§^	35.0 ± 7.1^§║^
RD‐Sham+Exos	8	100 ± 11^*§^	83 ± 5	800.0 ± 83.3^*§║^	10.7 ± 3.4^*║^
RD+PBS	9	87 ± 10^*#║^	81 ± 5	730.6 ± 71.9^§║^	13.8 ± 5.2^*║^
RD+Exos	10	89 ± 10^*#^	81 ± 7	1142.3 ± 181.6^*#†§║¶^	9.7 ± 2.1^*║^
90 days post‐MI					
RD‐Sham+PBS	5	116 ± 9^§║^	76 ± 6^§║¶^	598.4 ± 89.2^§^	26.8 ± 6.4^§║^
RD‐Sham+Exos	7	98 ± 10^*§║^	82 ± 5	754.3 ± 117.8^§║^	13.5 ± 5.6^║^
RD+PBS	8	88 ± 7^*#║^	81 ± 5	803.4 ± 95.4^§║^	13.3 ± 3.2^§║^
RD+Exos	10	84 ± 10^*#║¶^	79 ± 5	1409.0 ± 122.3^*#†§║¶^	8.4 ± 1.7^║^

*Note*: Continuous data are summarised as mean ± SD. Statistical comparisons utilised one‐way analysis of variance. *p *< .05: ^*^versus RD‐Sham+PBS, ^#^versus RD‐Sham+Exos, ^†^versus RD+PBS, ^§^versus baseline; ^║^versus on the day of MI, ^¶^versus 30 days after MI, ^$^versus 60 days after MI.

Abbreviations: Exos, exosomes; MI, myocardial infarction; PBS, phosphate‐buffered saline; RD, renal denervation.

### 3.4 Delayed exosomal therapy alleviates cardiac fibrosis by enhancing RD‐induced CM proliferation and regeneration

**FIGURE 5:**
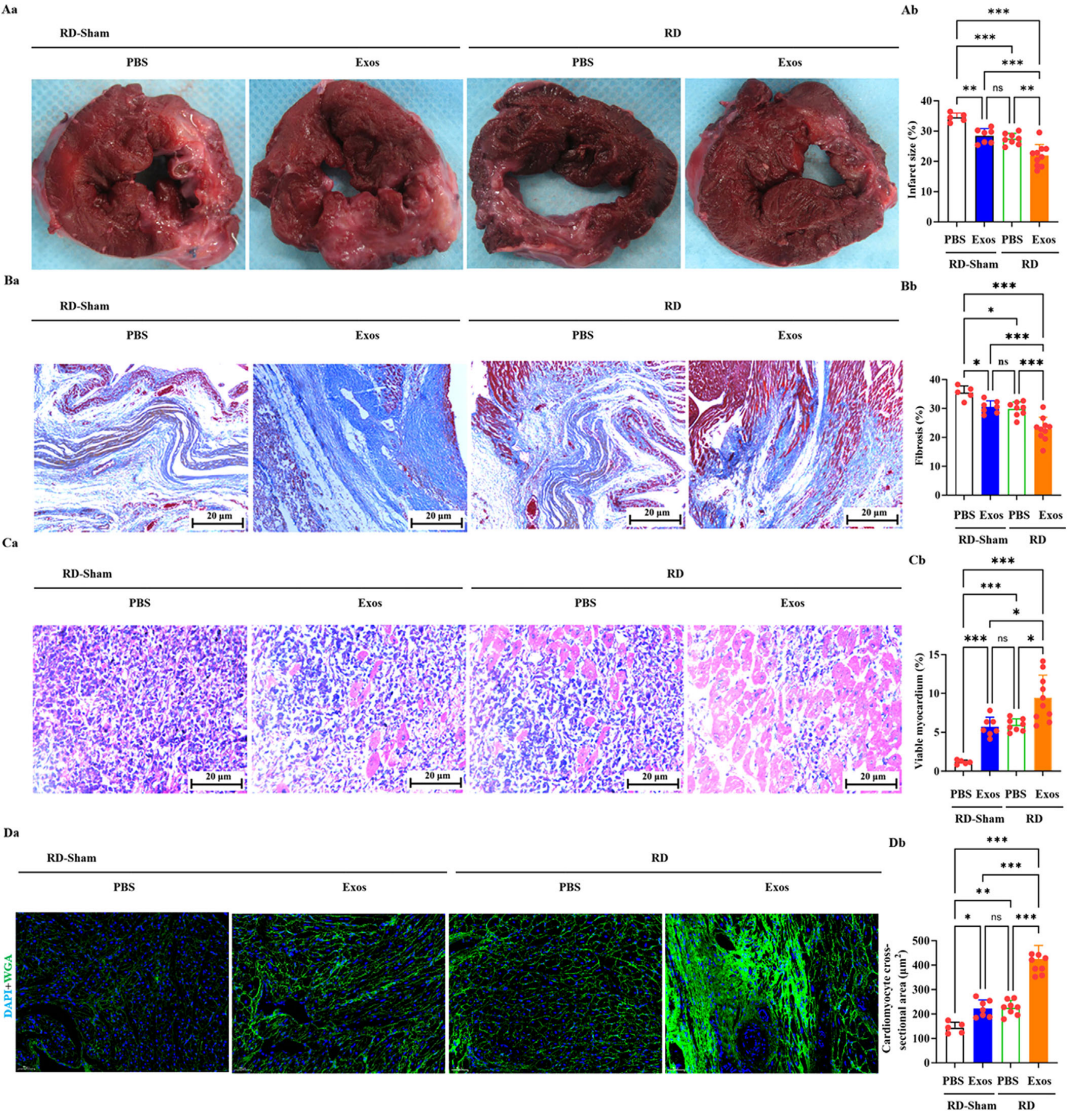
Renal denervation (RD) and delayed exosomal therapy improves pathological remodelling. (A‒C) Heart sections were stained subjected to triphenyl tetrazolium chloride (TTC) (A), Masson's trichrome (B), or haematoxylin‒eosin (H&E) (C) staining. Representative images (Aa) and quantification (Ab) of infarct size appearing pale white. Representative images (Ba) and quantification (Bb) of fibrosis (blue). Scale bar: 20 µm. Representative images (Ca) and quantification (Cb) of viable cardiomyocytes (CMs). Scale bar: 20 µm. (D) Wheat germ agglutinin (WGA) staining. (Da) Representative images of immunofluoroscence staining of WGA‐stained heart cross‐section of heart failure with reduced ejection fraction (HFrEF) pigs receiving RD‐Sham or RD in combination with or without delayed transplantation of peripheral blood mesenchymal stem cell (PBMSC)‐exosomes (Exos) 90 days after myocardial infarction (MI); nuclei (4′,6‐diamidino‐2‐phenylindole [DAPI]) appear blue, and WGA appears green. Scale bar: 50 µm. (Db) Graph showing cross‐sectional area measurements of cells showing WGA‐positive cytoplasmic localisation. All data, presented as means ± standard deviations, were analysed using one‐way analysis of variance. ^*^ *p *< .05, ^**^ *p *< .005, ^***^ *p *< .001; ns, non‐significant; *n* = 5 for RD‐Sham+PBS, *n* = 7 for RD‐Sham+Exos, *n* = 8 for RD‐Sham+PBS and *n* = 10 for RD+Exos. PBS, phosphate‐buffered saline.

We used triphenyl tetrazolium chloride, Masson's trichrome and haematoxylin‒eosin (H&E) staining for histological assessment 90 days after MI. Analysis revealed a marked reduction in scar size (Figure 5A) and fibrosis area (Figure 5B) in the RD+Exos group relative to all comparator groups. Moreover, this group exhibited the highest density of viable CMs (Figure 5C). Wheat germ agglutinin (WGA) staining was employed to quantify CMs cross‐sectional area. Both macroscopic postmortem analysis (Figure 5Da) and CM cross‐sectional area quantification (Figure 5Db) demonstrated that monotherapeutic administration of RD or Exos increased CM size, while co‐administration (RD+Exos) produced a further significant increase in cross‐sectional area at 90 days post‐MI. Comparable levels of these cardiac pathological remodelling indices were maintained in RD‐Sham+Exos and RD+PBS groups (NS). All these data suggest that both RD and Exos monotherapy inhibited cardiac pathological remodelling, and their combination therapy resulted in an enhanced therapeutic effect.

To decipher the cardioprotective mechanisms of RD plus staged Exos delivery against post‐MI remodelling, a tripartite experimental strategy was implemented: initial phase focused on circulating biomarkers of cardiac repair. Serial plasma collections at predefined intervals (baseline, 0/30/60/90 days post‐MI) enabled temporal quantification of Brain Natriuretic Peptide (BNP) and high‐sensitivity cardiac troponin T‌‌ (hs‐cTnT)di (Table 1). The results consistently indicated that RD can significantly increase plasma BNP levels and reduce plasma hs‐cTnT levels, and delayed exosomal therapy promoted these changes.

**FIGURE 6:**
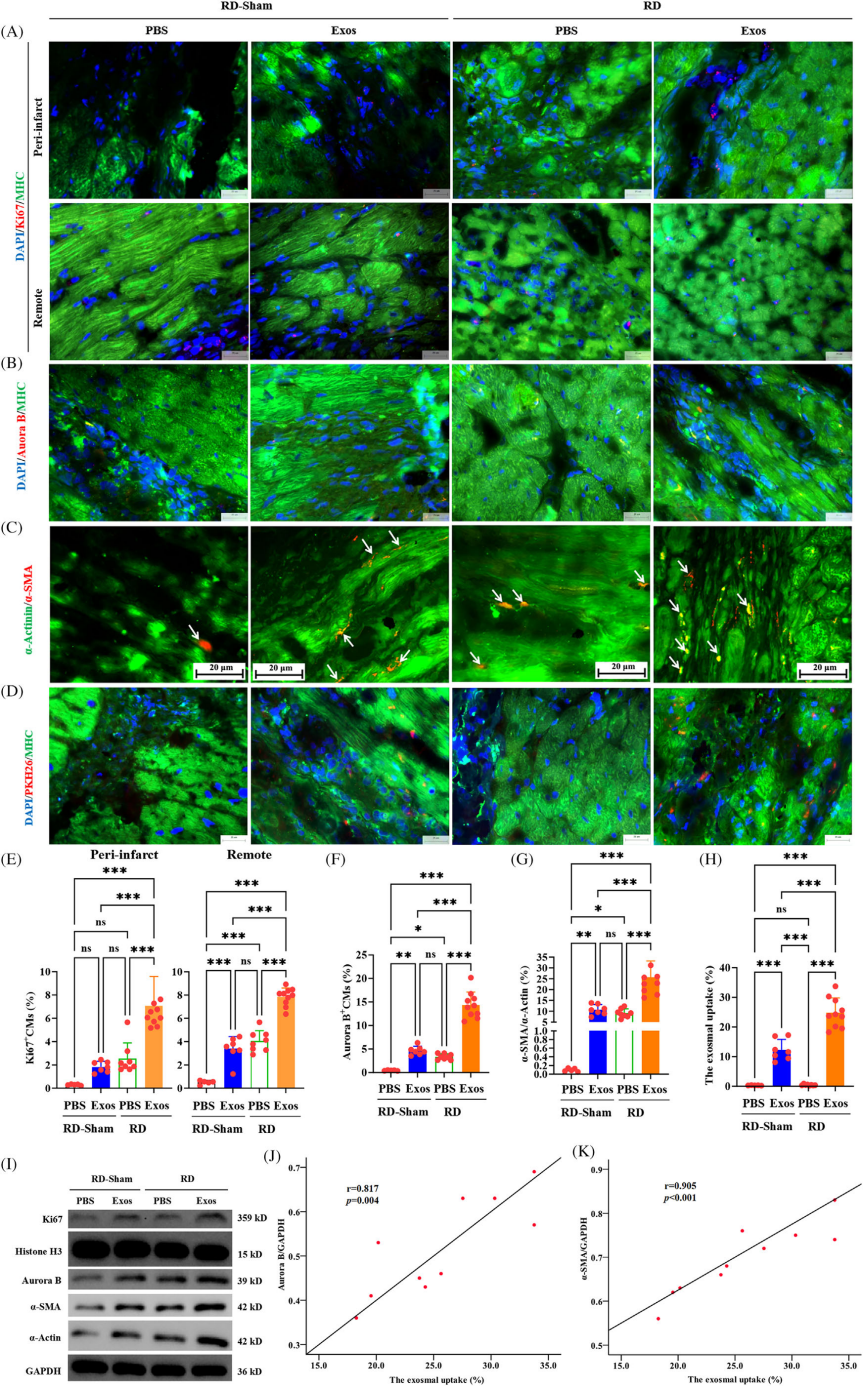
Exosomal therapy promotes renal denervation (RD)‐induced cardiomyocyte (CM) proliferation. (A) Representative images of immunofluorescence staining for Ki67 (red), myosin heavy chain (MHC) (green) and 4′,6‐diamidino‐2‐phenylindole (DAPI) (nuclei, blue) in the peri‐infarct (upper) and remote (lower) zone 90 days after myocardial infarction (MI), and its relative quantification (E). Scale bar: 25 µm. (B) Representative images of immunofluorescence staining for Aurora B (red), MHC (green) and DAPI (nuclei, blue) in the infarct borderzone 90 days after MI, and its relative quantification (F). Scale bar: 25 µm. (C) Immunofluorescence staining for α‐smooth muscle actin (α‐SMA) (red, marked by arrows) and α‐actin (green) in CMs from hearts cross‐sections. White arrows indicate related CMs c‐oexpressing α‐SMA. Scale bar: 20 µm. (G) Quantification of CMs dedifferentiation for immunofluorescence staining results in (C). (D and H) CMs uptake analysis of exosomes (Exos). At 90 days after MI, Exos were detected in myocardial tissues (MHC, red) of pigs with or without RD or exosomal therapy through immunofluorescence staining (D). Exos were pre‐labelled with PKH26 (red). Nuclei (DAPI) and MHC appear blue and green, respectively. Scale bar: 25 µm. (H) Quantification of CM uptake for Exos from immunofluorescence staining results in (D). All data, presented as means ± standard deviations, were analysed using one‐way analysis of variance. ^*^ *p *< .05, ^**^ *p *< .005, ^***^ *p *< .001; *n* = 5 for RD‐Sham+PBS, *n* = 7 for RD‐Sham+Exos, *n* = 8 for RD‐Sham+PBS and *n* = 10 for RD+Exos. (I) Western blotting for Ki67, Aurora B, α‐SMA and α‐actin expression were confirmed. (J and K) Relative correlation CM uptake for Exos with Aurora B (J) or α‐SMA (K) expression evaluated by Western blotting in the RD+Exos group (*n* = 10). PBS, phosphate‐buffered saline.

Second, CM proliferative activity was assessed following RD/Exos therapy. Ki67—an established proliferation marker expressed throughout active cell cycle phases (G1, S, G2, M)—served as the detection index, being absent during quiescence (G0).^27^ In the context of cardiac repair, Ki67 immunolabelling is used widely to assess CMs proliferation, which is a critical process for myocardial regeneration and functional recovery after injury.^28^ In this study, we used Ki67 immunolabelling to evaluate whether early RD (remote conditioning) and delayed Exos therapy could promote CM proliferation. Dual immunofluorescence co‐staining of Ki67 (proliferation marker) and myosin heavy chain (MHC, CM marker) demonstrated that early RD treatment significantly increased Ki67^+^ CM populations in both peri‐infarct and remote myocardial regions. Moreover, delayed Exos administration further enhanced this proliferative response, confirming the therapeutic potential of sequential interventions for CM cycle re‐activation (Figure 6A,E). Consistently, RD monotherapy upregulated Aurora B^+^ CMs density in myocardial tissues, while delayed Exos administration induced synergistic enhancement of this proliferative marker (Figure 6B,F). Subsequently, CM cell‐cycle reentry was validated via dedifferentiation analysis using α‐actin staining. RD monotherapy elevated α‐SMA^+^ CM prevalence, indicating partial dedifferentiation. This trend was markedly amplified in the RD+Exos cohort relative to RD‐Sham controls (*p* < .001) (Figure 6C,G). Western blotting confirmed that RD increased Ki67, Aurora B, α‐smooth muscle actin (α‐SMA) and α‐actin expression, and delayed Exos injection increased their expression further (Figure 6I).

Third, we assessed the uptake of PBMSC‐Exos by CMs in the RD‐Sham‐treated and RD‐treated hearts. To examine whether PBMSC‐Exos were differentially taken up by HFrEF hearts receiving RD‐Sham or RD, PKH26 red fluorescent dye was used to label the Exos before injection. PKH26 detection in the MHC through immunofluorescence staining (Figure 6D). PKH26 signals were absent in the CMs of the RD‐Sham+PBS and RD+PBS groups. However, strong PKH26 signals were observed in the CMs of RD‐Sham+Exos animals, and these signals were further enhanced by delayed Exos injection (Figure 6H). Striking correlations emerged between Exos internalisation efficiency and CM reprogramming markers: Aurora B expression (mitotic regulator; *r* = .817, *p *= .004; Figure 6J) and α‐SMA re‐expression (dedifferentiation index; *r* = .905, *p < *.001; Figure 6K) in RD+Exos‐treated hearts. Thus, RD‐induced proliferation and dedifferentiation of CMs may be related to the content and delivery of Exos, conferring regenerative capacity to an ischaemic heart.

### 3.5 Early RD alone reduces sympathetic nervous activation

**FIGURE 7:**
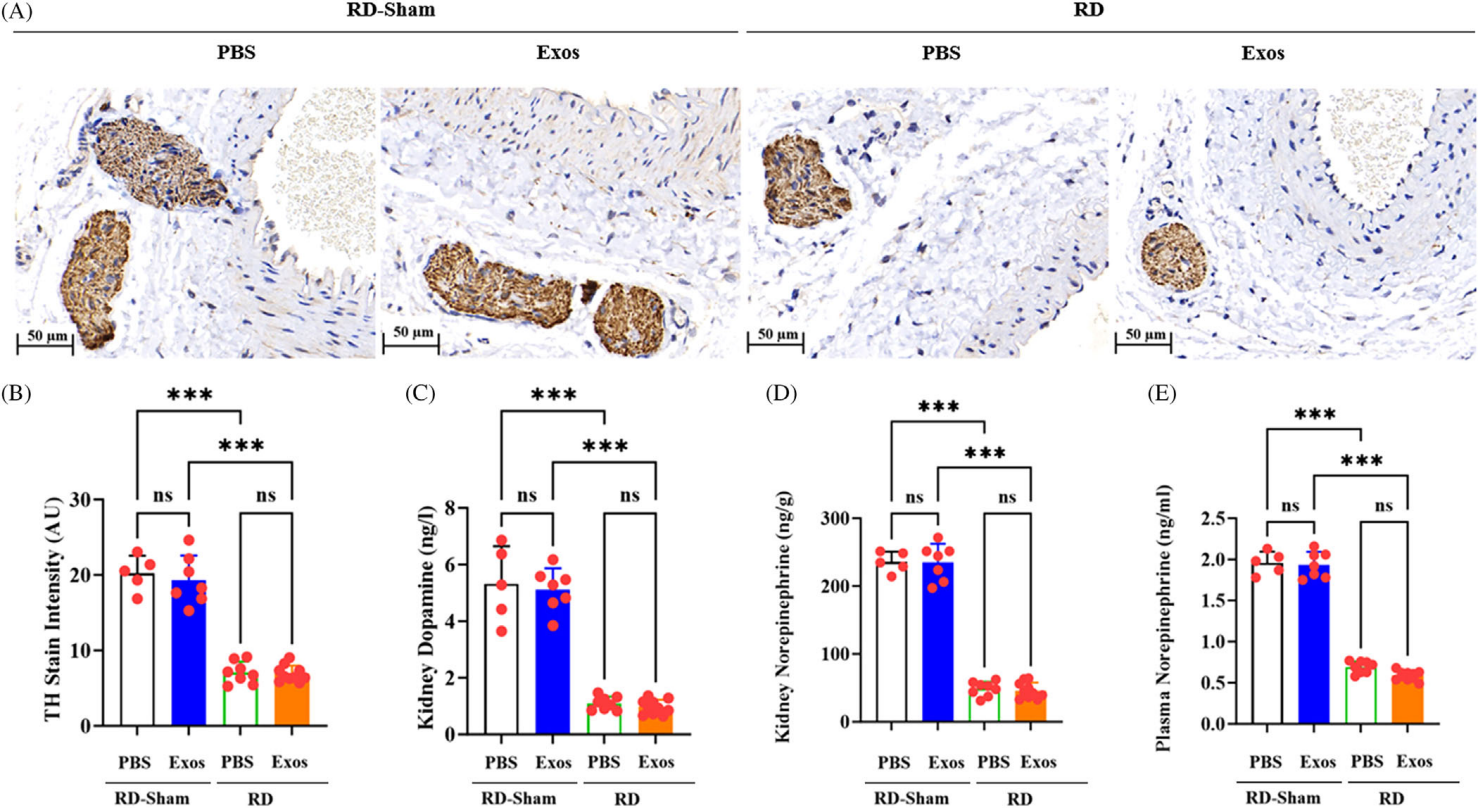
Renal sympathetic nervous system (SNS) activity in renal denervation (RD)‐Sham and RD pigs 90 days after myocardial infarction (MI). (A) Representative renal artery (RA) tyrosine hydroxylase (TH)‐stained photomicrographs from RD‐Sham pigs receiving phosphate‐buffered saline (PBS) or exosomes (Exos) and RD pigs receiving PBS or Exos. Scale bar: 50 µm. (B) TH staining intensity. (C and D) Kidney dopamine (C) and norepinephrine (NE) (D) concentration per gram of kidney cortex tissue. (E) Plasma NE levels 90 days after MI. All data, presented as means ± standard deviations, were analysed using one‐way analysis of variance.^***^ *p *< .001; ns, non‐significant. *n* = 5 for RD‐Sham+PBS, *n* = 7 for RD‐Sham+Exos, *n* = 8 for RD‐Sham+PBS and *n* = 10 for RD+Exos.

We next assessed the effects of early RD plus delayed exosomal therapy on the SNS of our pigs, specifically efferent renal nerve activity markers, including renal TH and NE. At 90 days post‐MI, immunohistochemical quantification revealed comparably attenuated TH staining intensity in both RD+PBS and RD+Exos groups relative to RD‐Sham+PBS and RD‐Sham+Exos controls (Figure 7A). Both RD+PBS and RD+Exos groups demonstrated an approximately 65% decrease in TH stain intensity compared with the RD‐Sham+PBS and RD‐Sham+Exos groups (*p *< .001 for each comparison; Figure 7B). Significant attenuation of renal SNS activity was evidenced by ∼80% reductions in kidney dopamine and NE levels at 90 days post‐MI in the RD+Exos group (*p *< .001 vs. baseline; Figure 7C,D). Concordantly, plasma NE decreased significantly and comparably in both the RD+PBS and RD+Exos groups compared to the RD‐Sham group (*p *< .001; Figure 7E). However, the lack of a significant difference in plasma NE levels between the RD+PBS and RD+Exos groups (*p* > .05) indicates that the additional cardioprotection conferred by delayed Exos therapy is largely independent of augmented SNS inhibition. This finding excludes SNS attenuation as the dominant mechanism underpinning the therapeutic benefits specific to delayed Exos administration.

### 3.6 Delayed exosomal therapy enhances RD‐induced RAAS inhibition

Like the aforementioned results, the RD+PBS and RD+Exos groups demonstrated significantly lower kidney angiotensin I and II levels at 90 days after MI than the RD‐Sham+PBS and RD‐Sham+Exos groups (Figure S3A,B). Moreover, RD‐induced alterations in angiotensin metabolism were reflected systemically, with significantly reduced plasma angiotensin I and II levels in both denervated groups (RD+PBS and RD+Exos) compared to their respective sham controls (RD‐Sham+PBS and RD‐Sham+Exos) (all *p *< .001; Figure S3D,E). Similarly, plasma renin activity decreased significantly in both RD+PBS versus RD‐Sham+PBS and RD+Exos versus RD‐Sham+Exos groups (both *p *< .001; Figure S3C). Kidney and plasma angiotensin I/II levels remained comparable between corresponding Sham and RD groups. Plasma aldosterone levels decreased significantly: RD groups were lower than their Sham counterparts (both *p *< .01), confirming renal denervation effect. Furthermore, Exos groups had lower aldosterone than PBS groups within both surgical conditions (RD‐Sham: *p *< .05; RD: *p *< .001), highlighting the specific efficacy of Exos therapy (Figure S3F).

### 3.7 PBMSC‐Exos^RD^ reprogram ischaemic CMs

The induction of adult CM proliferation is typically associated with three hallmark cellular transitions: (1) modulation of differentiation markers, (2) metabolic remodelling, and (3) re‐activation of embryonic gene programs. While this process historically has been termed ‘dedifferentiation’, emerging evidence supports its characterisation as ‘partial reprogramming’—a controlled transition to a foetal‐like proliferative state where adult CMs regain mitotic capacity while maintaining core functional characteristics.^29^ Notably, MSC‐Exos have been shown to orchestrate this regenerative process through their miRNA cargo, which simultaneously modulates tissue repair pathways and immune responses, thereby creating a permissive microenvironment for ischaemic myocardial regeneration.^30^ To comprehensively evaluate the effects of PBMSC‐Exos^RD^ enriched with the miR‐141‐200‐429 cluster on ischaemic CM reprogramming, we employed myocardial biopsies harvested 14 days post‐MI from RD‐Sham and RD‐treated animals (Figure 4A) within an integrated experimental framework. This strategy leveraged complementary in vitro and in vivo platforms for comprehensive assessment.

For in vitro functional assessment, CMs isolated from biopsied tissues of post‐MI hearts at 2 weeks were exposed to hypoxia‐mimicking conditions and cocultured with either PBMSC‐Exos^RD‐Sham^ or PBMSC‐Exos^RD^ (1 × 10^8^ particles/mL) for 48 h to systematically evaluate the composition‐dependent effects on CM functional recovery. The results demonstrated that 39.1% of CMs cultured with PBMSC‐Exos^RD‐Sham^ lacked cTnI, whereas this proportion significantly decreased in CMs cultured with PBMSC‐Exos^RD^, suggesting that PBMSC‐Exos^RD^ increased CM dedifferentiation (Figure S4A). Moreover, compared with PBMSC‐Exos^RD‐Sham^, PBMSC‐Exos^RD^ increased the levels of the dedifferentiation markers Runx1 and Dab2 (Figure S4B,C). Cell‐cycle reentry of CMs from PBMSC‐Exos^RD^‐treated CM cultures was confirmed by analysis of purified MHC‐expressing CMs positive for Ki67 (Figure S4Da) and PH3 (Figure S4Ea). Compared with CMs treated with PBMSC‐Exos^RD‐Sham^, all cases of CMs receiving PBMSC‐Exos^RD^ showed a much higher Ki67 proliferation index (Figure S4Db) concomitantly with an increased mitotic index (PH3^+^ cells; Figure S4Eb). Redifferentiation capacity, assessed by cTnT levels, was dramatically amplified (16.2‐fold increase vs. Sham; Figure S4F). These data provide compelling evidence that PBMSC‐Exos^RD^ uniquely facilitates the sequential steps of CM dedifferentiation, proliferation and functional redifferentiation under hypoxic conditions.

To evaluate in vivo therapeutic effects, we performed comprehensive transcriptional profiling of CMs isolated via cardiac biopsy from RD‐treated and RD‐Sham control groups at 14 days post‐MI, revealing significant PBMSC‐Exos^RD^‐mediated molecular reprogramming. Consistent with the functional and morphological assessments, these changes were related to cell proliferation, division and differentiation. Of the 10 genes significantly altered by PBMSC‐Exos^RD^ (Figure S5A), the expression of genes encoding six reprogramming factors involved with cardiovascular system development, cell specification, cycle, division and proliferation was upregulated, whereas four genes associated with inflammation and apoptosis were downregulated (Figure S5B). These findings collectively suggest that PBMSC‐Exos^RD^ treatment potentially induces molecular reprogramming in ischaemic CMs, facilitating their transition towards a regenerative state through activation of endogenous repair mechanisms.

### 3.8 β‐Catenin promotes Exos uptake, proliferation and redifferentiation by CMs

PBMSC‐Exos^RD^ were noted to reprogram CMs—as evidenced by overexpression of the pluripotent regulators Oct4, Sox2 and Klf4 (Figure S5A,B). Moreover, among all genes investigated, β‐catenin was the most significantly upregulated molecule by PBMSC‐Exos^RD^ (Figure S5B), suggesting that β‐catenin underlies CMs potency under hypoxic conditions.^31^

**FIGURE 8:**
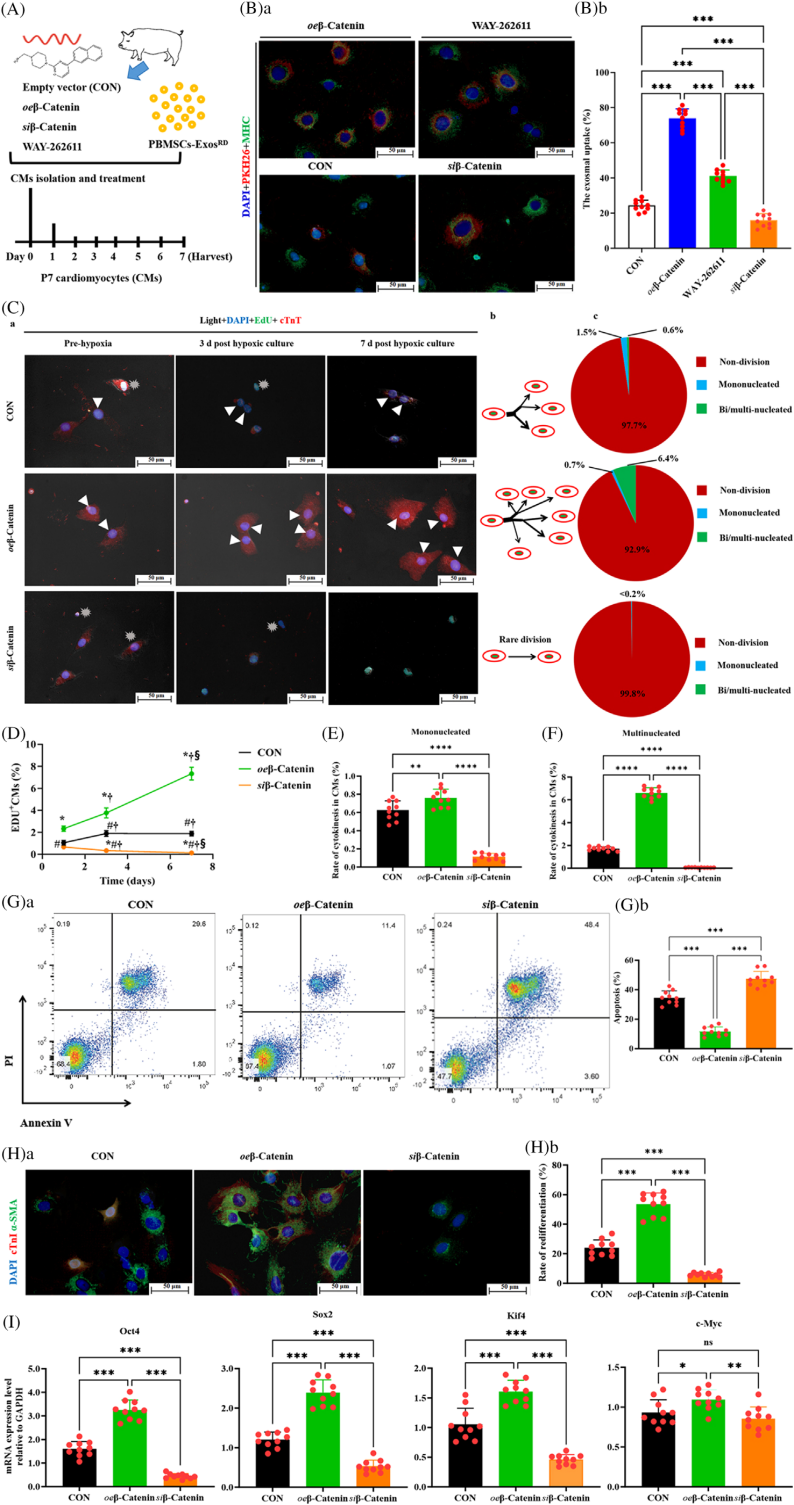
β‐Catenin promotes cardiomyocytes (CMs) exosomes (Exos)‐induced reprogramming. (A) Experimental design for investigating CM reprogramming in vitro. (B) CMs uptake rate of peripheral blood mesenchymal stem cell (PBMSC)‐Exos^RD^ evaluated by calculating proportion of cells expressing both PKH26 (red) and myosin heavy chain (MHC) (green) relative to all MHC‐positive cells. Representative images (Ba) and quantification (Bb) of CMs positively stained for PKH26 and MHC 7 days after coculture. Nuclei (4′,6‐diamidino‐2‐phenylindole [DAPI]) appear blue. Scale bar: 50 µm. (C) Identification of CM proliferation. (Ca) Morphological remodelling of CMs in the coculture system observed in a time‐relative manner. Binucleated cells among CMs treated with both PBMSC‐Exos^RD^ and *oe*β‐catenin (white arrows) became spherical during the first 3 days of coculture and then proliferated into several daughter cells over the next few days. Explosive symbols represent apoptotic cells. Nuclei (DAPI) appear blue. Scale bar: 50 µm. (Cb) Schematic of CM division patterns. (Cc) Mononucleated and binucleated or multinucleated CM proliferation rates. (D) Quantification curves of CMs expressing EdU at 1, 3 and 7 days after hypoxic coculture. *p *< .05: ^*^versus CON, ^#^versus *oe*β‐Catenin, ^†^1 day after hypoxic coculture, ^§^versus 3 days after hypoxic coculture (*n* = 10 each group). (E and F) Proliferation rates of mononucleated (E) and multinucleated (F) CMs. (G) Representative scatter plot (Ga) and apoptosis levels (Gb) of CMs assessed through flow cytometry after annexin V‒PI staining after 7 days of hypoxic culture. (H) Characteristics of dedifferentiated cells after CM cytokinesis immunostained for cardiac troponin I (cTnI) (red). (Ha) Representative images of CM‐derived dedifferentiated cells (α‐smooth muscle actin [α‐SMA], green) that regained sarcomeric structure (cTnI, red). Nuclei (DAPI) appeared blue. Scale bar: 50 µm. (Hb) Percentage of dedifferentiated cells with different properties. (I) Reprogramming factors’ mRNA expression levels in the CMs pretreated with empty vector, *oe*β‐catenin or *si*β‐catenin after induction with PBMSC‐Exos^RD^ for 7 days under hypoxic conditions. All data, presented as means ± standard deviations, were analysed using one‐way analysis of variance. ^*^ *p *< .05, ^**^ *p *< .005, ^***^ *p *< .001; ns, non‐significant (*n* = 10 each group) in (Bb), (E), (F), (Gb), (Hb) and (I). CON stands for transfection of empty vector.

Given the β‐catenin pathway's causally implicated role in CM differentiation,^32^, ^33^ we sought to delineate its contributory role in PBMSC‐Exo^RD^‐mediated CMs proliferation and redifferentiation. First, we assessed the β‐catenin‐promoted direct CMs uptake of MSC‐Exos. CMs were randomly transfected with an empty vector, *oe*β‐catenin, *si*β‐catenin or WAY‐262611 (1 µmol/L; a β‐catenin agonist; ab145229, Abcam) and then cocultured with PBMSC‐Exos^RD^ under hypoxic conditions (Figure 8A). To evaluate CM uptake efficiency, we calculated the proportion of PKH26^+^/MHC^+^ cells relative to total MHC^+^ cells (Figure 8Ba). Strikingly, β‐catenin overexpression (*oe*β‐catenin transfection) enhanced uptake most robustly, outperforming pharmacological activation (WAY‐262611) (Figure 8Bb). The least uptake levels were noted in *si*β‐catenin‐transfected CMs. Thus, β‐catenin may improve the Exos uptake of CMs. Since transfection with *oe*β‐catenin resulted in higher CM uptake compared to the β‐catenin agonist, we exclusively employed *oe*β‐catenin in the subsequent experiments.

Second, we determined whether β‐catenin induces a proliferative dedifferentiated state in PBMSC‐Exos^RD^‐treated CMs. CMs were pretreated with transfection of empty vector, *oe*β‐catenin, or *si*β‐catenin and then cultured for seven days with PBMSC‐Exos^RD^ under hypoxic conditions. We observed a considerable increase in CMs double‐positive for 5‐ethynyl‐2'‐deoxyuridine （EdU） and cTnT after *oe*β‐catenin transfection. After 7 days of culture, *oe*β‐catenin‐transfected CMs were rod‐shaped CMs, undergoing mitosis and cytokinesis (Figure 8Ca). Compared with empty vector transfection, *oe*β‐catenin transfection led to a continual increase in CM proliferation rates (Figure 8D): 7.1% of CMs proliferated (mononucleated:.7%; binucleated or multinucleated: 6.4%) after 7 days of culture. However, *si*β‐catenin transfection abrogated this benefit (Figure 8Cb–c). Multiple proliferation patterns were observed in the *oe*β‐catenin‐transfected cells. Binucleated CMs could divide into two or three mononucleated cells or into one mononucleated cell and one binucleated or multinucleated cell (Figure 8E,F). Thus, in the presence of PBMSC‐Exos^RD^, β‐catenin overcomes terminal differentiation barriers in CMs, inducing cell cycle reentry and proliferation across mononucleated and binucleated cells.

Third, β‐catenin dictated CM survival during hypoxic stress. Apoptosis rates (annexin V‒APC/PI flow cytometry; 7‐day hypoxia) were suppressed by β‐catenin overexpression but amplified by its knockdown—with *si*β‐catenin CMs showing maximal cell death (Figure 8G). Thus, β‐catenin may reduce CM apoptosis, promoting CM proliferation.

Fourth, after 7 days of culture, CMs were immunostained for cTnI to determine whether dedifferentiated CMs had redifferentiated (Figure 8Ha). Of the dedifferentiated CMs (i.e., α‐SMA‐positive CMs), the cTnI‐positive rates were 53.5%, 23.9% and 5.8% in *oe*β‐catenin‐transfected, empty vector‐transfected and *si*β‐catenin‐transfected CMs, respectively (Figure 8Hb). These results indicated that dedifferentiated CMs form new functional CMs.

Finally, β‐catenin potentiated Exos‐induced reprogramming. RT‐qPCR confirmed stronger induction of Yamanaka factors (*Oct4*, *Sox2*, *Klf4*, *c‐Myc*) in PBMSC‐Exo^RD^‐treated control CMs versus *si*β‐catenin CMs (*p *< .001; Figure 8I), aligning with prior protein data (Figure S5A). Strikingly, β‐catenin overexpression (*oe*β‐catenin) further amplified these transcripts, demonstrating its gatekeeper role in pluripotency activation.

### 3.9 PBMSC‐Exos^RD^ transfer miR‐141‐200‐429 clusters to induce heart regeneration via β‐catenin signalling

As established in our prior research, RD achieves cardioprotection by facilitating the release of Exos miR‐141‐200‐429 clusters from RAECs, which are transported to CMs through PBMSCs. We next probed whether miR‐141‐200c‐429 clusters serve as the molecular initiators of PBMSC‐Exos^RD^‐driven myocardial reprogramming through targeted activation of β‐catenin signalling.

**FIGURE 9:**
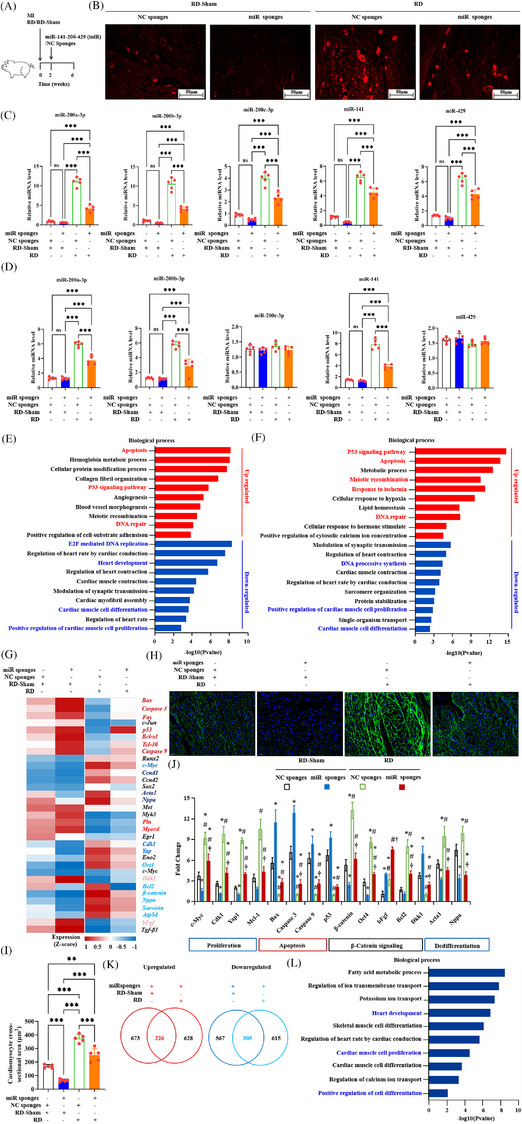
miR‐141‐200‐429 cluster abrogation inhibits renal denervation (RD)‐induced cardiac regeneration. (A) Schematic of protocol for miR‐141‐200‐429 or negative control (NC) sponge administration. Two weeks after RD or RD‐Sham, AAV9 injection was performed. (B) miR‐141‐200‐429 and NC sponges with endoglin‐adeno‐associated viral (AAV) were injected via the jugular vein. Thirty days later, fluorescence microscopy quantified BODIPY TR ceramide‐labelled exosomes (Exos) uptake in cardiac tissue. Scale bar: 50 µm. (C and D) Sponge‐mediated silencing of miR‐141‐200‐429 clusters in peripheral blood mesenchymal stem cell (PBMSC)‐Exos (C) and cardiomyocyte (CM)‐Exos (D) following renal artery endothelial cell (RAEC)‐targeted delivery (AAV9‐endoglin). ^***^ *p *< .001; ns, non‐significant (one‐way analysis of variance [ANOVA], Tukey's post hoc; *n* = 5). (E and F) Gene Ontology (GO) enrichment analysis of DEGs in CMs with RD‐Sham+miR‐141‐200‐429 sponges and RD+miR‐141‐200‐429 sponges. Enriched biological processes for upregulated and downregulated DEGs are shown in red and blue, respectively. (G) Heatmap of selected DEGs involved in apoptosis (deep red), contraction (red), nervous growth (light red), dedifferentiation (deep blue), proliferation (blue) and β‐catenin signalling (light blue). (H) Wheat germ agglutinin (WGA) immunofluoroscence staining of hearts receiving RD‐Sham, RD, NC sponges or miR‐141‐200‐429 sponges 44 days after myocardial infarction (MI); nuclei (4′,6‐diamidino‐2‐phenylindole [DAPI]) appear blue, and WGA appears green. Scale bar: 50 µm. (I) Quantitative hypertrophy assessment via WGA‐positive membrane delineation. Cross‐sectional areas shown as mean ± standard deviation (SD); ^*^ *p *< .05, ^***^ *p *< .001 (unpaired *t* tests with Holm‒Šidák correction; *n* = 5). (J) Real‐time quantitative polymerase chain reaction RT‐qPCR on selected genes in hearts of pigs receiving RD‐Sham, RD, NC sponges or miR‐141‐200‐429 sponges. Data, shown as mean ± SD, were analysed using one‐way ANOVA with Tukey's post hoc (*n* = 5 biological replicates/group). *p *< .05: ^*^versus RD‐Sham+NC sponges, ^#^versus RD‐Sham+miR‐141‐200‐429 sponges, ^†^versus RD+NC sponges. (K) Overlap of upregulated (left panel) and downregulated (right panel) DEGs in CMs with RD‐Sham and RD miR‐141‐200‐429 sponges compared with CMs with NC sponges. (L) GO enrichment analysis of DEGs from the overlap in (K).

To further confirm that the miR‐141‐200‐429 cluster detected in CMs originates from RD‐induced RAECs, we generated adeno‐associated viral (AAV) vectors expressing miRNA sponges targeting the miR‐141‐200‐429 cluster (‘miR sponge’) or NC sponges under the endoglin promoter (Figure 9A). Analysis of the miR‐141‐200‐429 cluster members revealed that robust myocardial enrichment was specific to miR‐200a‐3p, miR‐200b‐3p and miR‐141 (Figure 3G). Therefore, we constructed individual miR sponges targeting these three miRNAs for functional intervention experiments. Following the biopsy conducted at 14 days post‐MI, AAV therapy was immediately administered to the pigs treated with either RD‐Sham or RD. Four weeks later, PBMSC‐Exos were isolated, and CMs were collected. BODIPY TR ceramide‐labelled Exos uptake in hearts was detected through fluorescence microscopy. miR‐141‐200‐429 sponges significantly reduced CM uptake of these Exos in RD‐treated hearts (Figure 9B). These sponges significantly reduced miR‐141‐200‐429 levels in PBMSC‐Exos derived from the same amounts of cells (Figure 9C). Figure 9D shows that miR‐141‐200‐429 sponges effectively lowered miR‐200a‐3p, miR‐200b‐3p and miR‐141, but not miR‐200c‐3p or miR‐429, in CM‐Exos of RD pigs. This pattern of suppression confirms that the exosomal miR‐141‐200‐429 cluster present in CMs is specifically derived from RAECs subjected to RD.

RNA‐seq of CMs from the designated pig groups (RD‐Sham, RD‐Sham+miR‐141‐200‐429 sponge, RD+NC sponge, RD+miR‐141‐200‐429 sponge) was performed to confirm the miR‐141‐200‐429 cluster's role in heart regeneration. This analysis detected 1634 differentially expressed genes (DEGs) (*p* < .05) in the RD‐Sham+sponge group and 1745 DEGs (*p *< .05) in the RD+sponge group. Signalling pathways governing heart repair and regeneration are modulated by MSC‐derived Exos and their encapsulated miRNAs, highlighting their therapeutic potential.^24^, ^34^, ^35^ Gene set profiling showed miR‐141‐200‐429 sponges inhibited proliferative/differentiation gene programs but promoted apoptotic/contractile pathways, accompanied by decreased expression of dedifferentiation markers (Figure 9E‒G). Downregulated genes in proliferation/differentiation/cardiac development pathways reflect promoted CM maturation by miR‐141‐200‐429 sponges. This observation was further confirmed through WGA staining (Figure 9H) and CM cross‐sectional area quantification (Figure 9I), which confirmed a reduction in sarcomere density. Moreover, RT‐qPCR revealed increased expression of the proapoptotic genes *Bax*, *Casp3*, *Casp9* and *p53* but reduced that of immature CM‐specific genes involved in proliferation (i.e., *c‐Myc*, *Cdk1*, *Yap1* and *Mcl‐1*) and dedifferentiation markers (*Nppa* and *Acta1*) (Figure 9J) after miR‐141‐200‐429 sponge treatment. Integrative analysis of RNA‐seq data sets from RD‐Sham and RD miR‐141‐200‐429 sponge CMs revealed 226 and 305 genes jointly upregulated or downregulated in both HFrEF pig groups, respectively (Figure 9K). Most of the downregulated genes were those involved in heart development and CM proliferation and differentiation (Figure 9L). In summary, miR‐141‐200‐429 cluster disruption blocks RDCM dedifferentiation towards immaturity, resulting in suppressed proliferation and dedifferentiation capacity.

Of note, β‐catenin and its effector genes (*Bcl2*, *survivin*, *Oct4*) were significantly downregulated in RD+sponge group CMs, indicating concomitant inhibition of antiapoptotic signalling and cellular reprogramming compared to RD+NC sponges (Figure 9G,J). These findings suggest that miR‐141‐200‐429 sponges inactivates β‐catenin signalling, thereby suppressing a cascade of RD‐induced events that convert mature CMs into a more immature state, ultimately inhibiting CMs survival and reprogramming. However, we did not detect differences in the levels of the inflammation factor *Tgf‐β1* between RD+NC and RD+miR‐141‐200‐429 sponge group hearts, indicating that inhibition of miR‐141‐200‐429 cluster does not alter inflammation (Figure 9G).

Dickkopf‐related protein 1 (Dkk1), a suppressor of β‐catenin, and bFGF, a neurotrophic factor,^20^ were upregulated in miR‐141‐200‐429 sponge group CMs (Figure 9J). However, compared with RD+miR sponges, CMs receiving RD+NC sponges exhibited marked reductions in Dkk1 and bFgf expression (Figure 9J), indicating RAAS inhibition in RD‐treated hearts (Figure S3A‒F). β‐Catenin activation via Dkk1 suppression (induced by RD; Figure 9G,J) resulted in transcriptional downregulation of bFGF, a factor required for prorenin synthesis.^36^ This mechanism ultimately highlighted the beneficial effects of PBMSC‐Exos^RD^ related to RAAS inhibition.

To map functional targets of the RD‐PBMSC‐Exos‐derived miR‐141‐200‐429 cluster, we performed comprehensive loss‐ and gain‐of‐function screening. Given their cardiac enrichment (Figure 3G), miR‐200a‐3p, miR‐200b‐3p and miR‐141 were prioritised for mechanistic interrogation. RDCMs were then transfected with miRNA mimics (overexpression), miRNA‐specific sponges (inhibition), Scrambled oligonucleotides (mimic controls) or NC sponges (inhibition controls). After 48 h of hypoxic culture, qRT‐PCR analysis demonstrated that *Dkk1* mRNA levels were significantly decreased or increased with miRNA overexpression or inhibition, respectively (Figure S6A). Western blotting demonstrated an inverse correlation between miRNA levels and Dkk1 protein expression, with miRNA overexpression reducing and suppression increasing Dkk1 (Figure S6B). These findings provide evidence that the miR‐141‐200‐429 cluster directly targets Dkk1.

### 3.10 PBMSC‐Exos^RD^ enhance CM function via Dkk1 inhibition and β‐catenin activation

**FIGURE 10:**
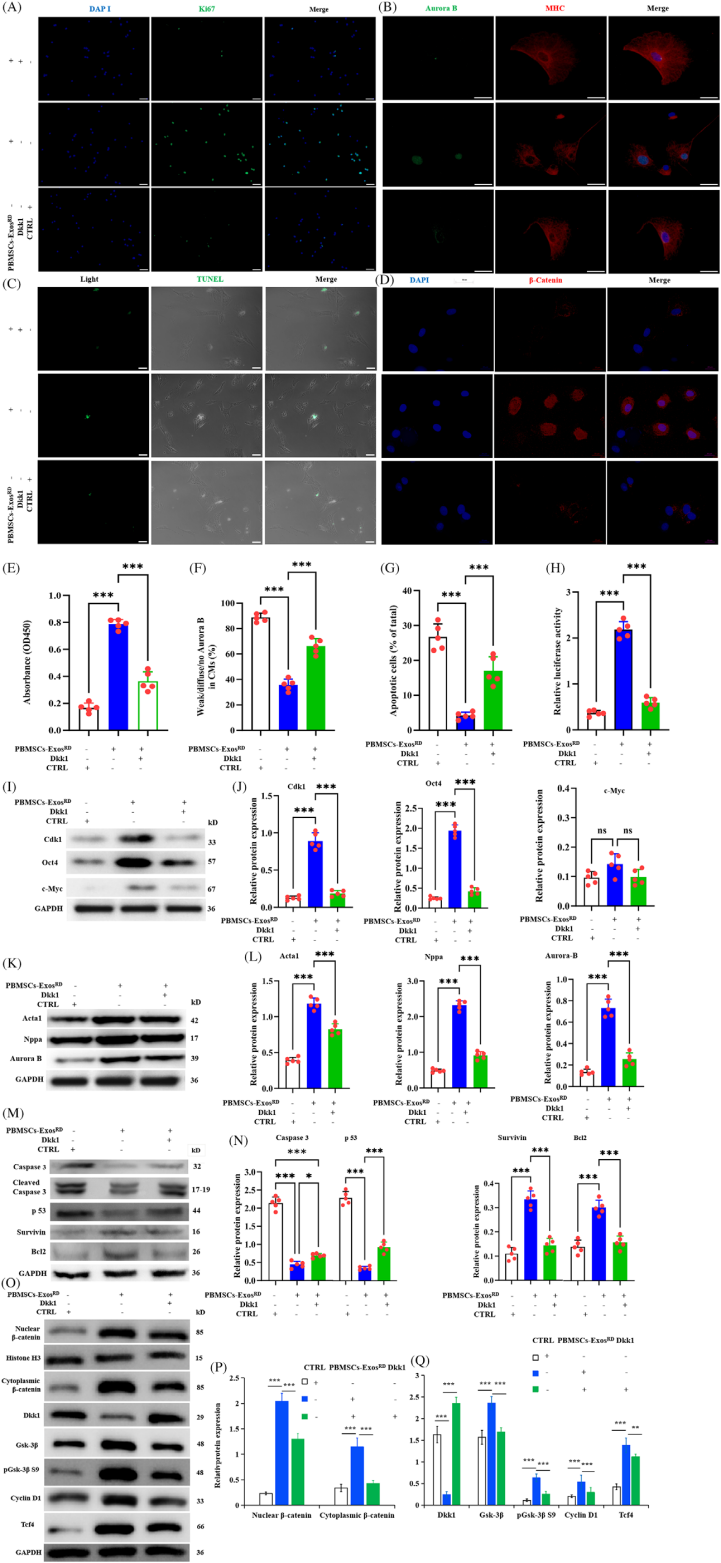
Peripheral blood mesenchymal stem cell (PBMSC)‐Exos^RD^ improve renal sympathetic denervation‐treated cardiomyocyte (RDCM) proliferation and dedifferentiation by targeting dickkopf‐1 (Dkk1). (A) Ki67 proliferation staining (50 µm). (B) Aurora B proliferation marker (50 µm). (C) TUNEL apoptosis assay (50 µm). (D) β‐Catenin localisation (20 µm). (E) Cell Counting Kit‐8 (CCK‐8) growth kinetics (OD450). (F) Quantification of results in (B) showing reduced proliferation in RDCMs cotreated with PBMSC‐Exos^RD^ and Dkk1, as indicated by the percentage of weak, diffuse or no Aurora B expression. (G) Apoptosis index quantification. (H) β‐Catenin transcriptional activity (luciferase). (I) Western blotting for Cdk1, Oct4 and c‐Myc in RDCMs treated with or without PBMSC‐Exos^RD^ or cotreated with PBMSC‐Exos^RD^ and Dkk1. (J) Cdk1/Oct4/c‐Myc quantification. (K) Western blotting for Acta1, Nppa and Aurora B in RDCMs. (L) Quantitative analysis of Acta1/Nppa/Aurora B. (M and N) Western blotting for Caspase‐3/p53/survivin/Bcl2 apoptotic modulation. (O‒Q) Wnt/β‐catenin axis dynamics: nuclear‐cytoplasmic β‐catenin, Dkk1, GSK‐3β signalling, cyclin D1. Data represent mean ± standard deviation (SD); ^**^ *p *< .01, ^***^ *p *< .001; ns, non‐significant (one‐way analysis of variance [ANOVA], Tukey's post hoc; *n* = 5 biological replicates). CTRL, control; DAPI, 4′,6‐diamidino‐2‐phenylindole; Exos, exosomes; MHC, myosin heavy chain; NC, negative control; RD, renal denervation.

Since miR‐141‐200‐429 sponges suppress β‐catenin activity and hinder CM rejuvenation by RD, we next asked if Dkk1 mediates these effects. To explore this, RDCMs were treated with PBMSC‐Exos^RD^ either alone or in combination with recombinant Dkk1. The RDCMs were then cultured for 7 days under hypoxic conditions to assess the effects. Although PBMSC‐Exos^RD^ ameliorated the proliferation of RDCMs, this benefit was abrogated when RDCMs cotreated with recombinant Dkk1, as demonstrated by Ki67 staining (Figure 10A) and Cell Counting Kit‐8 (CCK‐8) assay results (Figure 10E). Immunoblot analysis revealed that PBMSC‐Exos^RD^ enhanced Oct4 and the cyclin‐dependent kinase 1 (Cdk1) expression, while notably, c‐Myc expression remained largely unaltered (Figure 10I,J). However, Dkk1 overexpression reversed the PBMSC‐Exo^RD^‐induced enhancement of Aurora B incorporation (Figure 10B,F) and expression (Figure 10K,L), an enhancement previously observed in hearts treated with early RD followed by delayed Exos therapy (Figure 6B,F). Additionally, Dkk1 overexpression diminished the marked re‐expression of Nppa and Acta1 (Figure 10K,L)—both markers highly expressed during embryonic and foetal developmental stages.^37^ These findings suggest that Dkk1 disrupts the PBMSC‐Exos RD‐mediated activation of β‐catenin signalling, thereby negating its cardioprotective effects linked to RAAS inhibition and CM reprogramming. In contrast to these findings, TUNEL assay showed PBMSC‐Exo^RD^ significantly reduced CM apoptosis versus controls, an effect abolished by Dkk1 overexpression (Figure 10C,G). Dkk1 abolished PBMSC‐Exos^RD^‐mediated cytoprotection in CMs, evidenced by upregulated apoptotic executers (cleaved caspase‐3, p53) and downregulated survival factors (survivin, Bcl‐2) (Figure 10M,N), establishing that Dkk1 induces apoptosis in RD‐treated CMs to oppose therapeutic benefits; consequently, targeted Dkk1 inhibition represents a fundamental mechanism underlying PBMSC‐Exos^RD^’s cardioprotective action.

To confirm that RD‐induced β‐catenin activation is mediated by Dkk1 inhibition, we analysed β‐catenin‐associated protein expression in RDCMs receiving PBMSC‐Exos^RD^, recombinant Dkk1, or both, using Western blotting. The results demonstrated that PBMSC‐Exos^RD^ reduced Dkk1 expression while enhancing β‐catenin pathway activity—evidenced by increased p‐GSK‐3β (Ser9), cyclin D1, Tcf4 (Figure 10O,Q), and upregulated the pluripotency factor Oct4 (Figure 10I,J) relative to controls. Dkk1 cotreatment abolished PBMSC‐Exos^RD^‐mediated Wnt/β‐catenin activation and proliferative enhancement in RDCMs, confirming that exosomal benefits require Dkk1 repression. This aligns with our prior findings demonstrating that nuclear β‐catenin accumulation serves as a hallmark of canonical Wnt pathway activation.^16^ Developmental lung hypoxia induces pathological Wnt/β‐catenin activation via β‐catenin stabilisation. Subsequent nuclear accumulation occurs when GSK‐3β phosphorylation (e.g., at Ser9) inactivates this destruction complex kinase, enabling β‐catenin‐mediated transcriptional activation.^38^ Mirroring GSK‐3β phosphorylation dynamics, PBMSC‐Exos^RD^ elevated while Dkk1 suppressed β‐catenin accumulation—particularly in the nucleus—versus CTRL (Figure 10O,P). Immunofluorescence confirmed enhanced nuclear β‐catenin accumulation in PBMSC‐Exos^RD^‐treated cells versus CTRL, which was functionally suppressed by Dkk1 overexpression (Figure 10D,H). Collectively, these findings establish that miR‐141‐200‐429‐enriched PBMSC‐Exos^RD^ inhibits Dkk1—a key Wnt antagonist—triggering β‐catenin stabilisation via GSK‐3β inactivation. This cascade promotes β‐catenin nuclear translocation and transcriptional activation, reactivating the Wnt/β‐catenin pathway to enhance cardiac repair.

To establish whether Wnt/β‐catenin activation mediates RD+Exos‐induced reprogramming, we profiled key pathway effectors in myocardial tissue. As shown in Figure S7A,B, hearts from pigs treated with RD or Exos (PBMSC‐Exos^RD‐Sham^ or PBMSC‐Exos^RD^) exhibited elevated β‐catenin and p‐GSK‐3β (Ser9) levels versus the RD‐Sham+PBS controls. The combination treatments further amplified these expression levels relative to either treatment alone. Notably, both RD and Exos monotherapy significantly suppressed Dkk1 expression compared to PBS treatment in the RD‐Sham group, with the RD+Exos combination demonstrating an additive inhibitory effect on Dkk1. These findings are corroborated by immunohistochemical evidence (Figure S7C), demonstrating that RD and Exos both individually and combinatorially upregulate β‐catenin signalling through suppression of the pathway's negative regulator Dkk1.

Finally, to confirm that PBMSC‐Exo^RD^‐induced β‐catenin activation and subsequent CMs reprogramming contribute to heart regeneration, we employed a human pluripotent stem cell antibody array to assess the relative expression of β‐catenin transcriptional activation downstream targets^39^ in the hearts of pigs treated with RD‐Sham+PBS, RD‐Sham+Exos, RD+PBS or RD+Exos. The expression of three crucial pluripotency factors (i.e., Oct4, Klf4 and Nanog), two β‐catenin nuclear retention factors (i.e., YAP1 and TCF4),^40^ and a cardiac lineage marker (i.e., Gata4) were upregulated in both RD+PBS and RD‐Sham+Exos groups, peaking significantly in the RD+Exos cohort (Figure S7D). In contrast, hearts of pigs receiving the combination treatment exhibited significantly reduced levels of β‐catenin destruction regulators LEF1 and Axin2 (Figure S7E).^41^ Expression of other Wnt/β‐catenin‐associated factors—including SMAD2, Sox2, c‐Myc, Wnt2 and TCF7—remained comparable across all experimental groups (Figure S7E). These findings were corroborated by immunoblot assays, which revealed that hearts from pigs co‐treated with RD and Exos displayed the highest expression of Oct4, Klf4, Nanog and Gata4 but the lowest expression of LEF1 and Axin2 (Figure S7F), suggesting a reprogramming state in HF hearts following RD+Exos treatment. Immunohistochemical staining further validated these results, demonstrating increased density of the cardiac β‐catenin‐relative reprogramming transcription factors Oct4, Klf4, Nanog, YAP1, TCF4 and Gata4 (Figure S7G).

## 4 DISCUSSION

Our study establishes that RD combined with PBMSC‐derived Exos enhance cardiac reprogramming and functional recovery in a porcine MI model. This repair mechanism operates through miR‐141‐200‐429‐dependent suppression of Dkk1, reactivating the Wnt/β‐catenin pathway to promote CM plasticity. Key findings include: (1) PBMSC‐derived Exos demonstrated therapeutic efficacy through enhanced functional recovery and infarct size reduction; (2) miR‐141‐200‐429 clusters within Exos drove the reprogramming of mature CMs into a more immature, regenerative state; and (3) this reprogramming was mechanistically dependent on Dkk1 inhibition and subsequent β‐catenin upregulation, as demonstrated by rescue experiments using AAV‐delivered miR‐141‐200‐429 sponges. These results highlight a novel therapeutic strategy targeting Wnt/β‐catenin signalling to mitigate post‐MI remodelling.

RD was initially developed as a treatment for resistant hypertension. Given the sympathoinhibitory effects of RD, Polhemus et al. demonstrated that RD represents a novel therapeutic strategy to reduce SNS activation and enhance left ventricular performance, with benefits extending beyond blood pressure lowering.^42^ Building on this, we explored the remote, direct cardioprotective effects of RD on CMs. To address the therapeutic gap in proliferative MI repair, we evaluated RD‐mediated functional improvement in HFrEF‐MI porcine hearts. Our findings revealed that elevated SNS activity under MI conditions exacerbates myocardial ischaemic stress, suppresses proliferation signalling and activates the apoptosis pathway. In contrast, RD‐treated pigs exhibited a significant reduction in kidney SNS activity. Post‐MI RD administration enhanced cardiac function, improving LVEF while normalising end‐systolic/diastolic volumes and attenuating pathological LV remodelling. However, this local attenuation of SNS activity was transient, lasting only up to 2 weeks post‐MI, and did not sustain long‐term, sustained improvements in cardiac function, remodelling or survival rates (Figure 4). These results underscore the need to enhance the remote cardioprotective effects of RD to achieve sustained cardiac repair and improved outcomes following MI‐induced HF.^42^

In the current study, we identified five novel findings. First, we provided direct evidence that RD‐derived PBMSC‐Exos therapy promotes the beneficial effects of RD on LV performance, resulting in long‐term, sustained improvement in the cardiac outcomes of our HFrEF pig model (Figure 4). The clinical application of RD for hypertension treatment remains controversial. Furthermore, it is still unclear whether RD exerts lasting effects or can be applied effectively to other SNS‐related conditions, such as HF, atrial fibrillation and sleep apnoea syndrome.^43^ Our HFrEF pig model revealed that the therapeutic impact of RD on SNS and RAAS activity was stage and time dependent. Marked suppression of both systems was achieved in RD‐treated pigs, reaching its zenith at the 2‐week mark following MI (Figure 1). In addition to disrupting renal afferent signalling pathways, RD demonstrated significant attenuation of ventricular fibrosis (Figure 5B) and downregulation of myocardial pro‐inflammatory mediators (interleukin‐6 and nuclear factor‐κB) (Figure S5A,B), which effectively mitigate macrophage‐driven fibrotic cascades.^44^ These findings suggest RD exerts regulatory effects on ventricular fibrosis progression in post‐MI remodelling. In contrast, the release of PBMSC‐Exos increased significantly in a time‐dependent manner, peaking 2 weeks after MI in the RD group. However, the magnitude of these changes diminished during subsequent follow‐up time. This observation may explain why the simplicity HTN‐3 trial results failed to demonstrate further improvement in clinical outcomes 6 months after RD.^45^ Delayed MSC‐Exos administration resulted in sustained HF‐preventive effects in RD pigs, persisting up to 90 days post‐MI. Specifically, LV function and dimensions were preserved, infarct expansion was suppressed, and post‐MI survival rates significantly improved. These findings provide critical insights into potential therapeutic approaches for HFrEF (Figures 4 and 5). Figures 4 and 5 demonstrate that the RD‐Sham+Exos and RD+PBS groups exhibited equivalent cardiac structure, function and pathological remodelling at 90 days post‐MI. This functional equivalence demonstrates that PBMSC‐Exos from RD pigs fully replicate the therapeutic benefits of RD monotherapy. The findings suggest that RD's long‐term cardioreparative effects are mediated primarily via Exos secretion, rather than by direct attenuation of SNS activity. Consequently, the outcomes achieved through the exogenous administration of PBMSC‐Exos (RD‐Sham+Exos) are functionally equivalent to those induced by endogenous Exos release following RD treatment (RD+PBS). Furthermore, the relative improvement magnitude was consistent across models: RD‐Sham+Exos (Exos therapy alone) ≈ RD+PBS (RD monotherapy). This parallelism confirms PBMSC‐Exos as the principal mechanistic drivers of RD‐induced cardiac repair. Critically, combining RD with exogenous exosomes (RD+Exos) synergistically amplified reparative capacity beyond either monotherapy, conclusively underscoring Exos as the primary effector mechanism of RD‐mediated cardiac repair.

Second, we obtained the first evidence that PBMSC‐Exos^RD^ induce reprogramming of ischaemic CMs. Several recent studies have indicated that RD may have beneficial effects on ventricular remodelling in post‐MI HF.^8^, ^42^ These findings confirm that RD elicits multifaceted cardioprotective effects in HF, primarily through robust attenuation of renal SNS/RAAS hyperactivation, concomitant suppression of oxidative stress and GRK2 activity, and enhanced nitric oxide signalling. Nevertheless, the precise mechanistic pathways by which RD improves clinical outcomes remain incompletely characterised in preclinical HFrEF models and constitute an ongoing focus of investigation.^46^ In the current study, we observed transient SNS and RAAS activity‐lowering effects in HFrEF pigs; however, this attenuation could not be sustained without any significant changes in plasma and kidney SNS and RAAS activity 15 days after MI (Figures 1 and 7). As such, these short‐term effects cannot sufficiently explain long‐term improvements in cardiac function after RD+Exos. We then focused on the other mechanisms through which Exos may promote cardiac repair by preserving LV function independent of the attenuation of kidney SNS and RAAS activities. Thus far, Exos have been successfully employed to augment the survival efficacy and differentiation capacity of human‐induced pluripotent stem cells (hiPSCs), promoting their maturation into functional CMs.^47^ Intercellular communication mediated by Exos constitutes a significant mechanism underpinning post‐MI myocardial repair and associated signalling transduction processes.^48^ Here, we present the first preclinical assessment of PBMSC‐Exos^RD^ in a large animal model of HFrEF following MI. The increased proliferation of CMs in RD‐treated hearts, Exos‐treated hearts and PBMSC‐Exos^RD^‐treated CM cultures was demonstrated through Ki67 (Figures 6A and S4D) and pH3 (Figure S4E) staining‐established biomarkers for cell cycle activity. This observed proliferation indicates enhanced CMs division capacity, a crucial mechanism for myocardial regeneration.^49^ As shown in Figure 6A,E, Ki67^+^ CM counts rose significantly in peri‐infarct/remote zones with early RD monotherapy relative to RD‐Sham. This suggests that RD stimulates endogenous regenerative mechanisms, including CMs proliferation. Delayed Exos injection further enhanced this effect, leading to a greater number of Ki67‐positive CMs in the RD+Exos group. This additive interaction suggests that delayed Exos therapy amplifies the regenerative benefits of early RD.

Terminally differentiated CMs cultured with PBMSC‐Exos^RD^ reentered the cell cycle and proliferated under hypoxic conditions, with CM proliferation occurring through redifferentiation under hypoxia (Figure S4F). Consistent with our immunofluorescence staining results, gene detection analysis revealed that PBMSC‐Exos^RD^ upregulated the expression of factors related to proliferation (*Cdk1* and *Yap1*) and reprogramming (*Oct4*, *Sox2* and *Klf4*) (Figure S5). Similar findings were noted in our in vivo animal study. In HFrEF pigs, RD enhanced mature CM proliferation and dedifferentiation, and subsequent delayed exosomal therapy further amplified these effects (Figure 6A‒C). These functional outcomes correlated with histomorphometric analyses showing significantly reduced scar burden and fibrosis—consistent with the greatest increase in viable CM density (Figure 5C). This is consistent with Gallet et al.’s observation that cardiosphere‐derived cell Exos reduce scarring, mitigate adverse remodelling and improve LVEF in HFrEF pigs.^50^ CM Exos internalisation was significantly elevated in RD‐treated HFrEF pigs compared to RD‐Sham counterparts (Figure 2A‒E). Notably, RD enhanced CM uptake of Exos, and delayed exosomal therapy further increased this uptake in HFrEF pigs (Figure 6D,H). These findings were corroborated by recent studies showing a robust increase in the Exos level in the blood of RD‐treated mice.^31^ Additionally, Exos uptake was positively correlative with cardiac proliferation and dedifferentiation in the HFrEF pigs receiving the combination of RD and exosomal therapy. These findings collectively elucidate RD‐mediated inhibition of LV remodelling in HFrEF porcine models and reveal novel therapeutic targets: sustaining circulating Exos levels to preserve their cardioprotective functions—a strategy with potential to reduce post‐MI mortality.

Third, β‐catenin emerged as the central regulator of RD‐mediated myocardial reprogramming. Critically, cardiorenal‐derived Exos modulate proangiogenic paracrine signalling in adipose‐derived MSCs post‐MI—revealing their previously unrecognised role in cardiorenal syndrome pathophysiology.^51^ In newly established mouse models, endothelial‐derived caveolin‐1^+^ Exos traffic to adipocytes—revealing a previously unrecognised inter‐tissue communication pathway.^52^ We pioneer the observation that RD triggers RAEC secretion of functional PBMSC‐Exos, which traffic through circulation and localise to damaged CM (Figure 3). Moreover, RD significantly upregulates the myocardial expression of β‐catenin (Figure S5)—a major signal transduction molecule in MSC‐Exos widely involved in regeneration or repair‐related biological processes.^53^ This finding aligns with our observation that RD‐treated CMs exhibited a marked upregulation of reprogramming factors *Oct4*, *Sox2* and *Klf4* (Figure S5), a phenomenon associated with β‐catenin activation.^54^ Notably, β‐catenin overexpression led to a continuous increase in CMs uptake of PBMSC‐Exos^RD^ and proliferation, redifferentiation and apoptosis prevention in hypoxia‐cultured CMs (Figure 8). Within tumor microenvironments, Exos shuttle oncogenic cargo (e.g., β‐catenin, CEACAM1, HER2, Melan‐A/MART‐1, LMP1) bidirectionally between malignant cells and stromal compartments, reprogramming recipient cells through horizontal oncogene transfer.^55^ Taken together, these findings further highlighted the importance of β‐catenin upregulation by circulating PBMSC‐Exos for RD‐mediated myocardial reprogramming.

Fourth, we identified the miR‐141‐200‐429 cluster as the most essential molecule in myocardial reprogramming induced by PBMSC‐Exos^RD^. Emerging evidence reveals exosomal miRNAs as master regulators of tissue regeneration and immune homeostasis.^56^ Rodent models establish that RD attenuates LV fibrosis through miRNA‐dependent pathways, revealing a key mechanistic axis for its therapeutic efficacy.^57^ As such, we performed high‐throughput screening and found that PBMSC‐Exos^RD^ contained higher levels of miR‐141‐200‐429 clusters than PBMSC‐Exos^RD‐Sham^ did (Figure 3D). miR‐141‐200‐429 clusters are generated by RD‐treated RAECs and carried by PBMSCs from RAECs to CMs (Figure 3E). All five miR‐141‐200‐429 cluster genes were significantly upregulated in RAECs (Figure 3F), while only miR‐200a‐3p, miR‐200b‐3p and miR‐141 showed significant elevation in CMs (Figure 3G). RAEC‐specific delivery of miR‐141‐200‐429 sponges to both RD‐Sham and RD pigs substantially impaired CM exosomal uptake in infarcted hearts (Figure 9B) and abolished RD‐induced upregulation of miR‐200a‐3p/miR‐200b‐3p/miR‐141 in treated CMs (Figure 9D). Notably, these sponges significantly suppressed β‐catenin‐associated pathways governing proliferation, apoptosis and dedifferentiation (Figure 9G,J). Exos‐encapsulated miRNAs enhance CMs proliferation,^58^ with certain human miRNAs exhibiting cross‐species capacity to promote rodent CM cell cycle reentry and cardiac regeneration post‐MI.^59^ Collectively, these findings elucidate a novel RD‐mediated positive feedback loop wherein exosomal miRNA transfer amplifies myocardial repair mechanisms: RD promotes the release and uptake of Exos rich in miRNAs. miRNAs then enhance the remote cardioprotective effects of RD, improving CM proliferation, antiapoptosis and regeneration, as well as systolic LV function through activation of β‐catenin signalling under HF conditions (Graphical Abstract). However, further validation through additional studies, including clinical studies, is essential to confirm these findings and explore the potential therapeutic applications. Finally, through integrated bioinformatic analysis and subsequent multi‐faceted experimental validation, we identified *Dkk1* (Dickkopf‐related protein 1) as a target gene of the novel miR‐141‐200‐429 cluster. Dkk1, a secreted inhibitor of the Wnt/β‐catenin signalling pathway critical for cell proliferation, differentiation and survival,^60^ is known to be targeted by various miRNAs. These miRNAs silence *Dkk1* during early osteogenic differentiation; however, *Dkk1* expression becomes upregulated in later stages as miRNA levels decline.^61^ Crucially, we establish for the first time that direct targeting of *Dkk1* by the miR‐141‐200‐429 cluster, and its consequent inhibition following RD, is a pivotal regulator of CMs proliferation and redifferentiation. Since miR‐141‐200‐429 sponges inhibit the β‐catenin pathway and hinder the RD‐induced reprogramming pathways (Figure 9E,F), it is essential to investigate whether Dkk1, as an inhibitor of this pathway, plays a regulatory role in these processes. By analysing Dkk1, we have gained insights into multiple mechanisms through which it modulates the effects of miR‐141‐200‐429 sponges on Wnt/β‐catenin signalling pathway and the consequent CM reprogramming (Figure 9G,J), including: (1) preventing CM proliferation and expression of its markers c‐Myc, Mcl‐1, Yap1 and Cdk1; (2) preventing CM dedifferentiation and expression of its markers Nppa and Acta1; and (3) increasing CM apoptosis and expressions of the apoptotic proteins Bax, caspase3 and p53 along with decreased expression of antiapoptotic proteins survivin and Bcl2. Moreover, Dkk1 overexpression was found to significantly suppress the PBMSC‐Exos^RD^‐mediated upregulation of phosphorylated GSK‐3β, β‐catenin nuclear translocation, as well as the expression of cyclin D1 and Tcf4 (Figure 10O‒Q), which are miR‐200a targets.^62^ Building on established evidence that Dkk1 pretreatment blocks Wnt1‐driven differentiation via GSK‐3β/β‐catenin axis suppression,^63^ and attenuates target gene induction (*cyclin D1*, *Tcf4*, *Lef1*).^64^ We conceptualise Dkk1 as a master regulatory node in cellular reprogramming. Notably, our study uncovers a previously unrecognised exosomal signalling cascade: RAEC miR‐141‐200‐429 clusters are enriched in PBMSC‐Exos and exosomally shuttled to recipient CMs. Within CMs, this cluster executes targeted *Dkk1* silencing—dismantling a key β‐catenin brake—and thereby initiates β‐catenin‐driven transcriptional reprogramming (Figure 9G,J). Increasing PBMSC‐Exos production, enhancing CMs biogenesis, or activating β‐catenin target genes (particularly Tcf4) (Figure 10O,Q) may be novel, effective therapeutic avenues to improve PBMSC‐Exos‐mediated beneficent communication between RAs and MI hearts after RD, enhancing cardiac regeneration in acute MI (Figure S8). Taken together, these results suggested that PBMSC‐Exos participate in the protective effects of RD against ischaemic injury.

Current studies demonstrate that the post‐MI inflammatory response progressively transitions into the reparative phase, followed by the remodelling phase.^65^ Therefore, the treatment for MI must address both acute‐phase anti‐inflammatory interventions and chronic‐phase management of myocardial fibrosis. This study demonstrates that the sequential therapeutic approach integrating acute‐phase RD intervention with later‐phase Exos administration achieves comprehensive functional recovery in infarcted hearts throughout the post‐MI repair process. This translation aligns with current guidelines emphasising stage‐specific treatment strategies in MI.

We also found that delayed Exos injection significantly reduced plasma aldosterone levels (Figure S3). It also increased circulating BNP levels sustainably with RD (Table 1). RD‐induced increases in circulating BNP led to improvements in cardiac function and protection against ischaemia‒reperfusion injury,^31^ suggesting that long‐term cardioprotective effects may arise from the additive actions of RAAS inhibition and enhanced cardiac function.

### 4.1 Study limitations

While our findings demonstrate the potential of PBMSC‐Exos miRNAs to enhance cardiac repair of RD after MI, this study has several limitations. First, early cardiac biopsy specimens collected at 14 days post‐MI from RD‐treated and RD‐Sham hearts demonstrated that PBMSC‐Exos^RD^ significantly upregulated pluripotency transcription factors (Oct4, Klf4, Sox2) in RDCMs (Figure S5). However, longitudinal analysis revealed no detectable increase in Sox2 or c‐Myc expression in ischaemic cardiac tissues from either group, even at the 90‐day post‐MI endpoint (Figure S7). This temporal discrepancy—where transient transcriptional activation occurs acutely but dissipates chronically—may reflect dynamic shifts in epigenetic regulation, microenvironmental crosstalk (e.g., inflammatory or fibrotic signalling) or unresolved compensatory feedback loops. While these findings underscore the context‐dependent nature of cellular reprogramming, their clinical extrapolation necessitates rigorous consideration of spatiotemporal biological constraints and interspecies translational gaps. Second, while in vitro analyses identified β‐catenin as a pivotal regulator of hypoxia‐induced endogenous reprogramming in mature mammalian CMs, we were unable to corroborate this mechanism through functional gain‐ or loss‐of‐function experiments, such as β‐catenin overexpression or knockdown in vivo. The physiological fidelity of our pathway mechanism remains constrained pending in vivo verification, where systemic crosstalk and tissue‐level integration critically modulate Wnt/β‐catenin dynamics. Third, while in vitro sponge‐mediated miR‐141‐200‐429 knockdown blocked RA‐Exos’ cardioprotection under hypoxia, validation in an MI model via RA‐Exos‐specific miR‐141‐200‐429 overexpression is lacking. Future studies employing targeted activation of miR‐141‐200‐429 in RA‐Exos, followed by functional assessments in MI models, would clarify its specific role and enhance the translational relevance of our findings. Fourth, while this study identified key miRNAs in RD‐Exos that promote cardiac repair, the lack of comparative RNA‐seq data for RD‐Sham‐Exos limits our understanding of molecular differences between these Exos populations. Future studies should address this gap to fully elucidate mechanisms underlying their functional divergence. Fifth, our study did not evaluate RD‐Sham‐derived Exos in RD animals. While our data demonstrate the efficacy of RD‐derived PBMSC Exos in RD pathology, this limitation prevents definitive conclusions about whether their enhanced therapeutic effects—compared to sham‐derived Exos in RD animals—stem from inherent molecular differences or microenvironment interactions. Future reciprocal transplantation studies (e.g., sham‐derived Exos in RD models and RD‐derived Exos in sham models) will clarify this mechanistic distinction.

## 5 CONCLUSIONS

We establish RD as a novel therapeutic strategy conferring ischaemia‒reperfusion protection in large mammalian HFrEF post‐MI, mediated through targeted potentiation of endogenous PBMSC‐Exos delivery systems enriched with miR‐141‐200‐429 regulatory networks. These miRNAs orchestrate multiphasic cardioprotection by attenuating neurohormonal activation (reduced circulating catecholamines and suppressed RAAS activity), inhibiting apoptosis pathways (downregulation of Bax/Caspase‐3), enhancing myocardial stress adaptation (elevated BNP expression) and activating β‐catenin‐mediated proliferative signalling.

Notably, the RD+Exos combinatorial strategy enabled sustained miRNA persistence (up to 90 days) in ischaemic myocardium, overcoming the transient bioavailability limitations of conventional miRNA therapies. While RD's therapeutic potential was initially attributed to blood pressure modulation, our findings reveal broader mechanistic implications, including epigenetic reprogramming and paracrine crosstalk. This positions RD not merely as a haemodynamic intervention but as a platform for targeted organ protection, warranting exploration in other chronic conditions characterised by oxidative stress and maladaptive remodelling (e.g., diabetic cardiomyopathy, chronic kidney disease). Furthermore, we propose a translational framework integrating RD with precision medicine approaches: (1) temporal synergy: co‐administration with guideline‐directed antihypertensive therapies to optimise haemodynamic and cellular repair phases; (2) spatial control: nanoparticle engineering of Exos for tissue‐specific miRNA delivery; (3) safety escalation: biomarker‐guided titration to minimise off‐target epigenetic effects. These preclinical insights bridge the gap between observational cardioprotection and mechanism‐driven therapeutic innovation, offering a roadmap for clinical translation in HFrEF management.

## AUTHOR CONTRIBUTIONS

*Conceptualisation, methodology, writing—original draft preparation and preparing* *Figures* *1* *‒* *5*: Lan Zhao. *Supplement data for later revisions*: Chen Li. *Data curation and preparing* *Figures* *6* *‒* *10*: Zhichuan Huang. *Visualisation, investigation and preparing* *Table* *1*: Jianshuo Wang. *Supervision and preparing* Figures *S1‒S6*: Zhanyu Deng. *Software, validation and preparing* Figure *S7* *and* Tables *S1 and S2*: Shaoheng Zhang. *Preparing* Figure *S8* *and Graphical Abstract*: Yanwen Deng. *Writing—reviewing and editing*: Shaoheng Zhang. All the authors reviewed the manuscript.

## CONFLICT OF INTEREST STATEMENT

The authors declare they have no conflicts of interest.

## ETHICS STATEMENT

The Institute for Animal Care and Use Committee at Dahua Hospital approved all the animal experiments, which were carried out in compliance with the Guide for the Care and Use of Laboratory Animals published by the National Academies Press (http://www.nap.edu/).

## CONSENT FOR PUBLICATION

All authors have read and approved the final manuscript.

## Supporting information



Supporting Information

Supporting Information

Supporting Information

Supporting Information

Supporting Information

Supporting Information

Supporting Information

Supporting Information

Supporting Information

Supporting Information

Supporting Information

Supporting Information

## Data Availability

The datasets used and/or analysed during the current study are available from the corresponding author upon reasonable request.
