# Supplementary figures and images for: Peripheral blood mesenchymal stem cell‐derived exosomes improve renal sympathetic denervation efficacy through β‐catenin‐mediated cardiac reprogramming
Source: Clin Transl Med. 2025 Sep 5;15(9):e70475. doi: 10.1002/ctm2.70475 (PMC12411928; doi:10.1002/ctm2.70475)

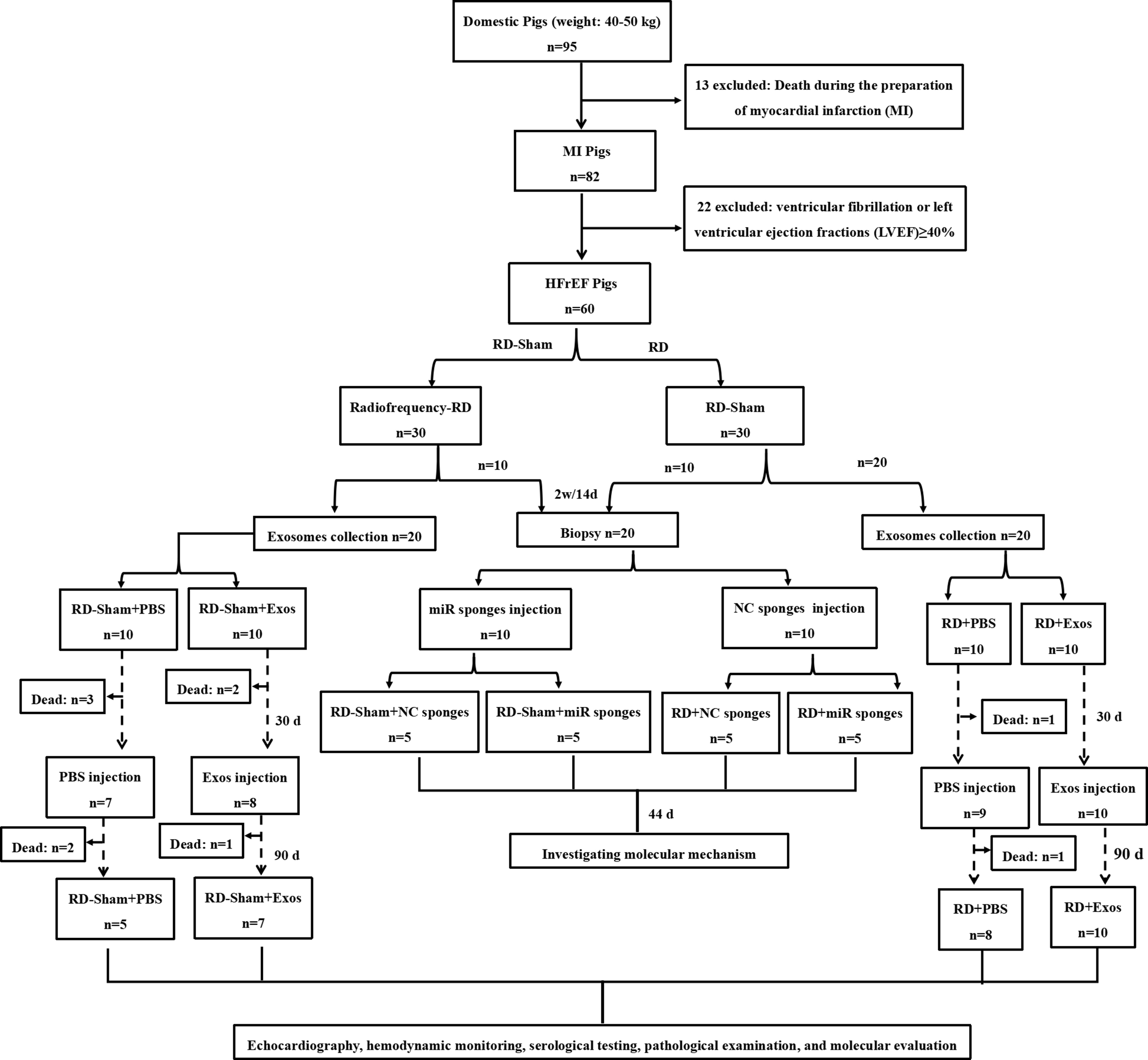

Supplement: Supplementary file 1 — Supporting Information [file CTM2-15-e70475-s007.jpg]

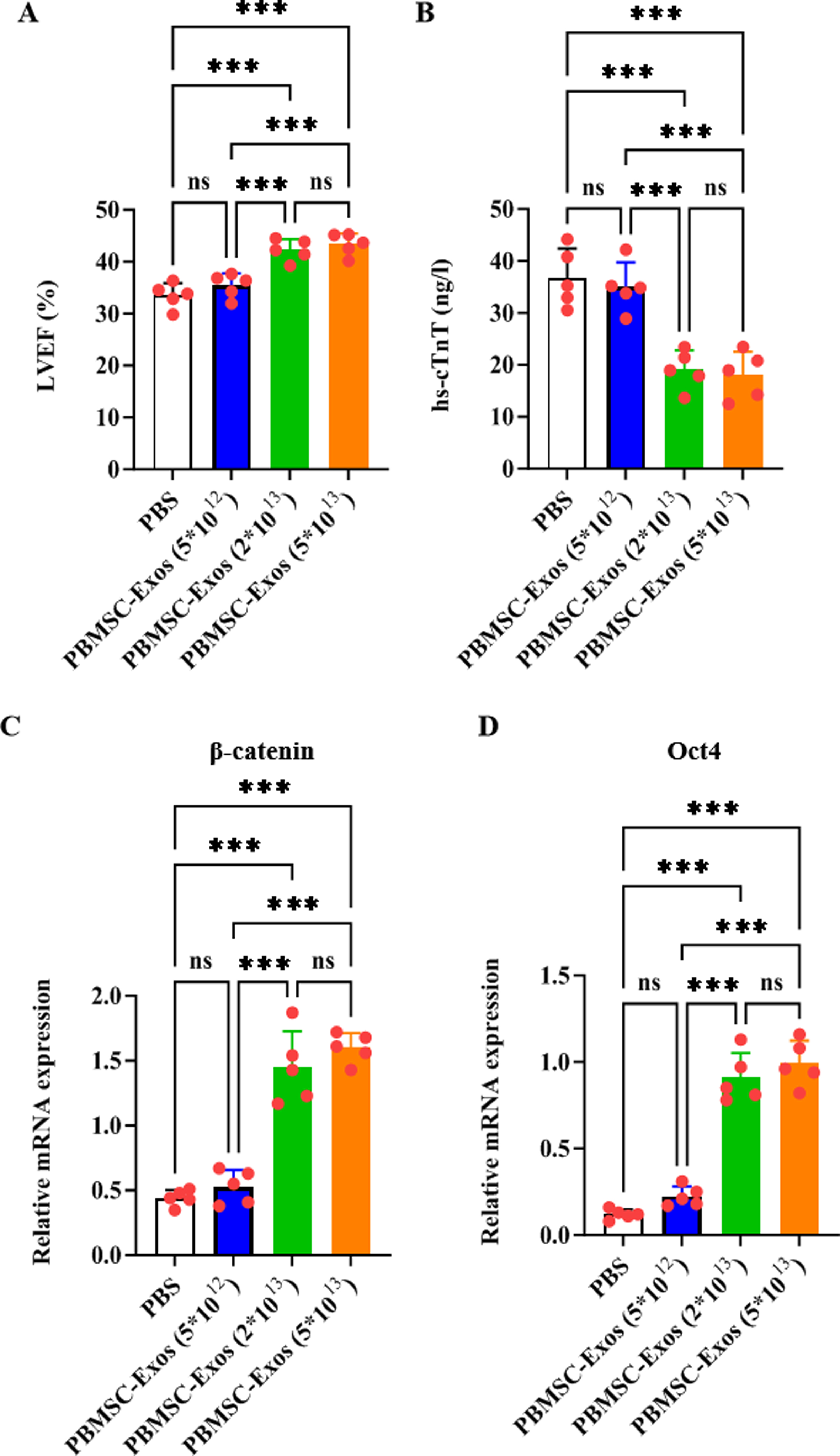

Supplement: Supplementary file 2 — Supporting Information [file CTM2-15-e70475-s010.jpg]

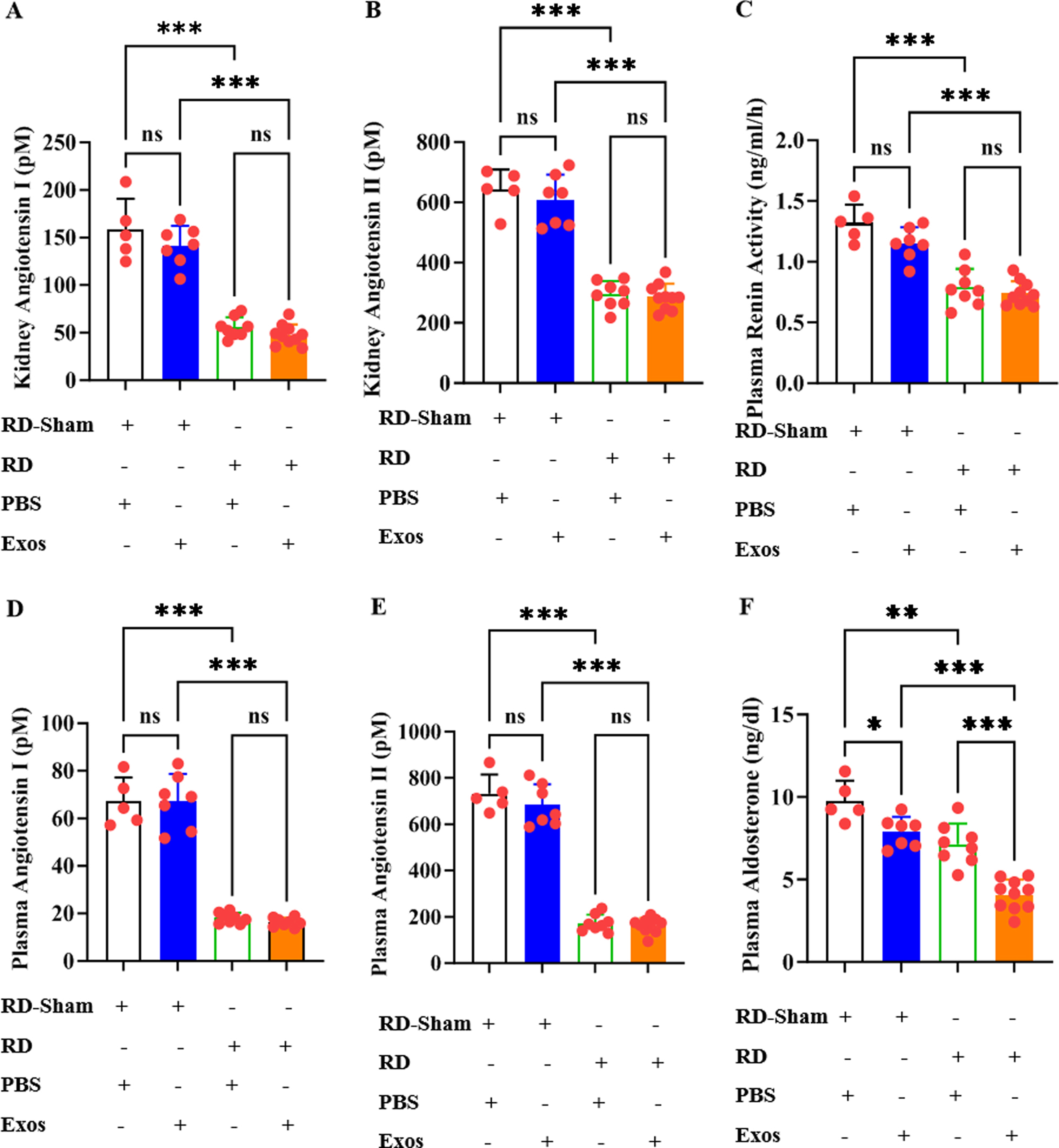

Supplement: Supplementary file 3 — Supporting Information [file CTM2-15-e70475-s006.jpg]

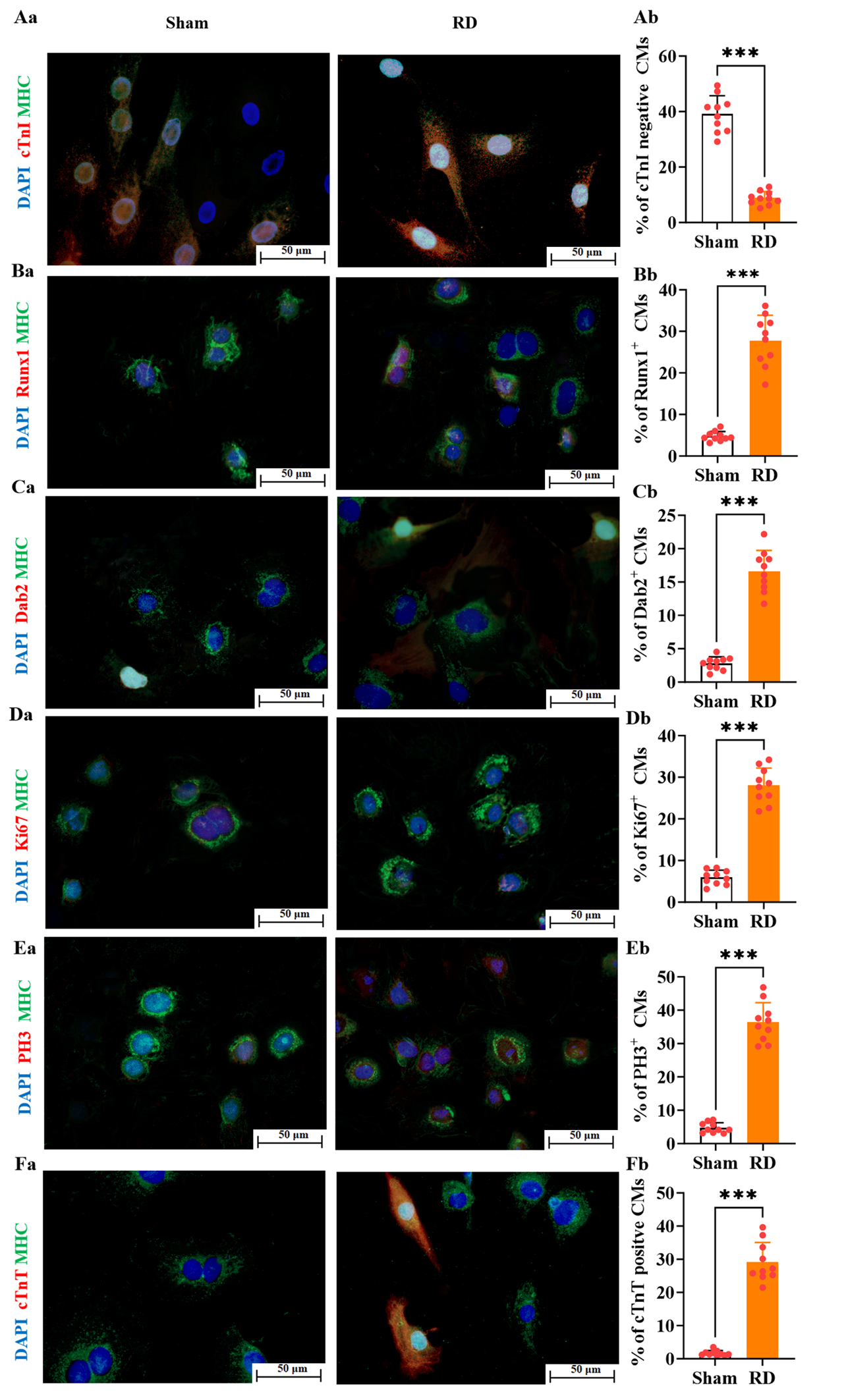

Supplement: Supplementary file 4 — Supporting Information [file CTM2-15-e70475-s003.jpg]

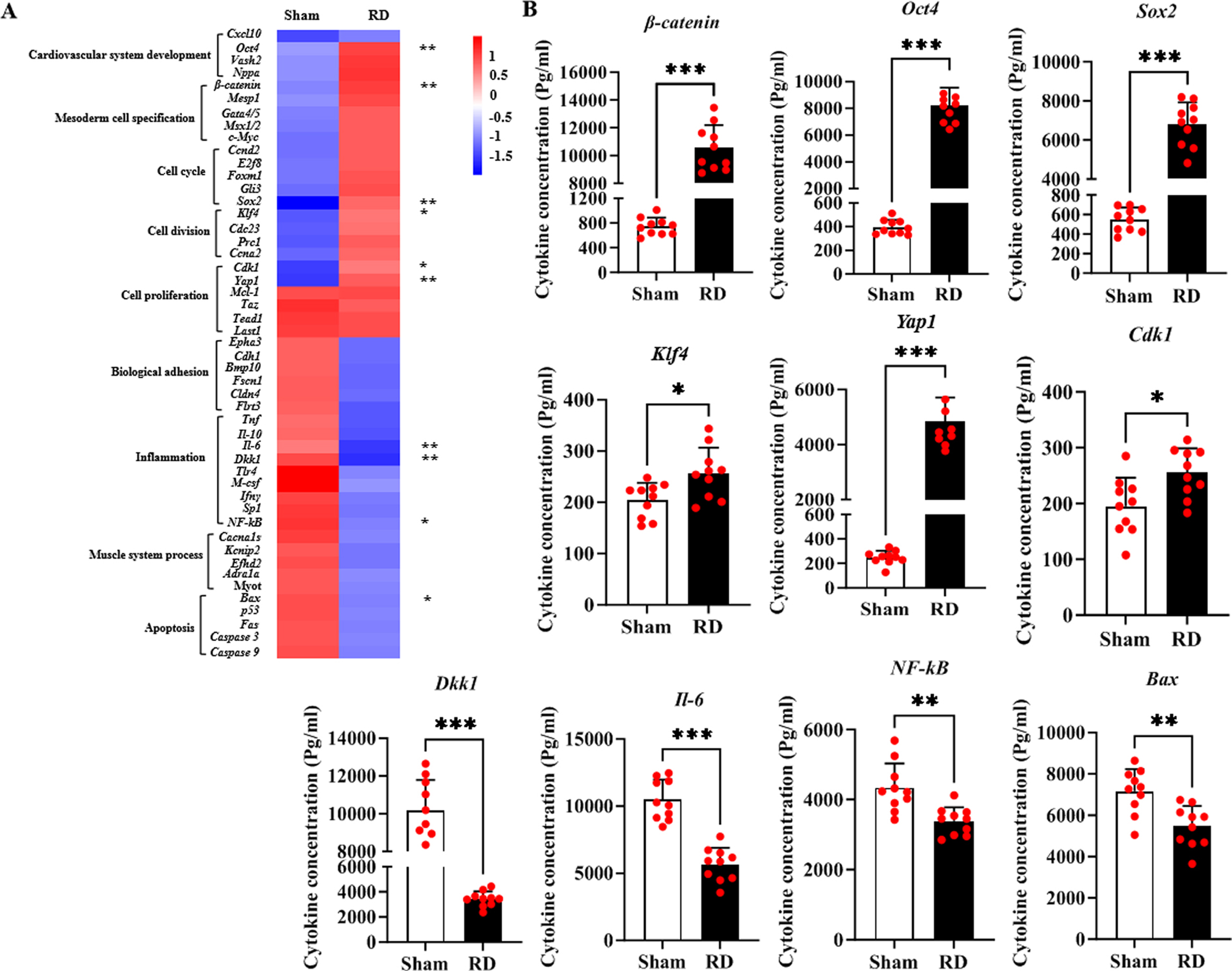

Supplement: Supplementary file 5 — Supporting Information [file CTM2-15-e70475-s005.jpg]

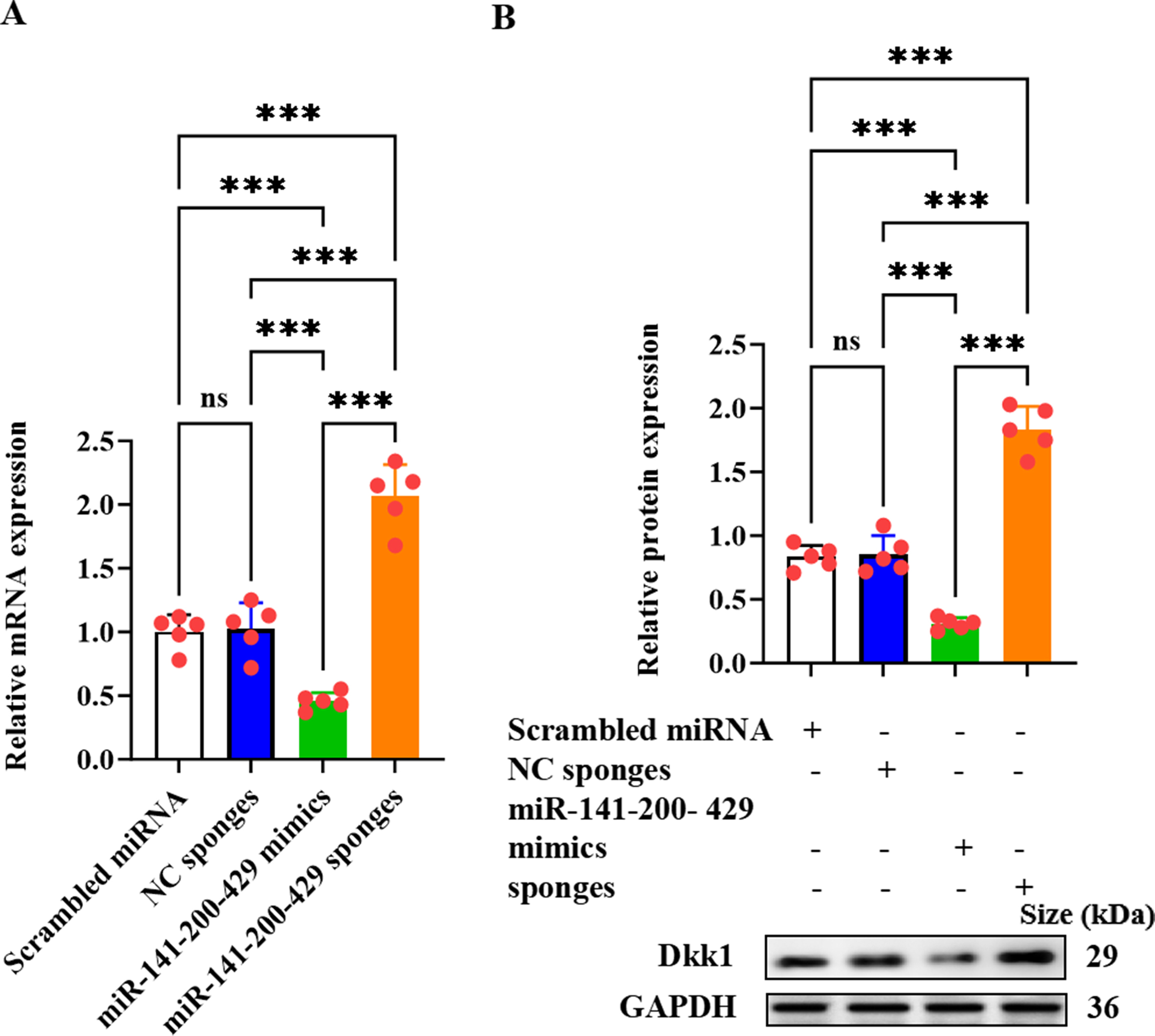

Supplement: Supplementary file 6 — Supporting Information [file CTM2-15-e70475-s011.jpg]

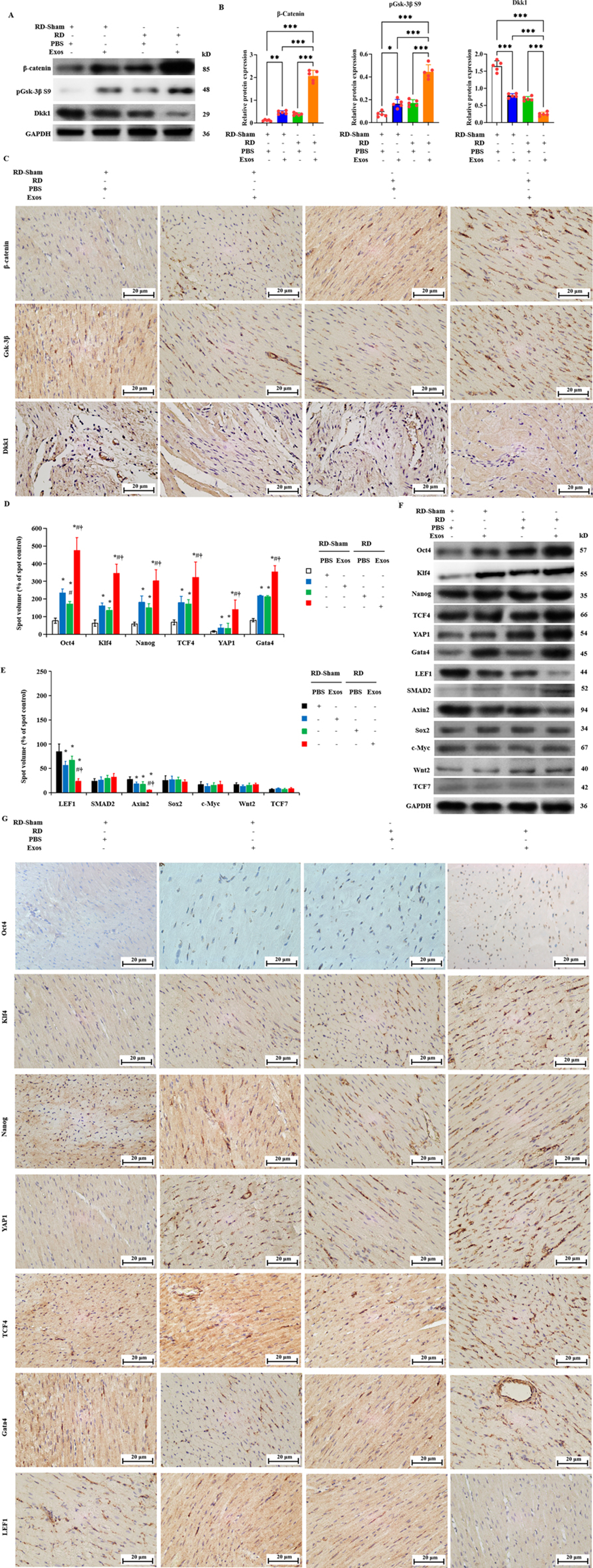

Supplement: Supplementary file 7 — Supporting Information [file CTM2-15-e70475-s012.jpg]

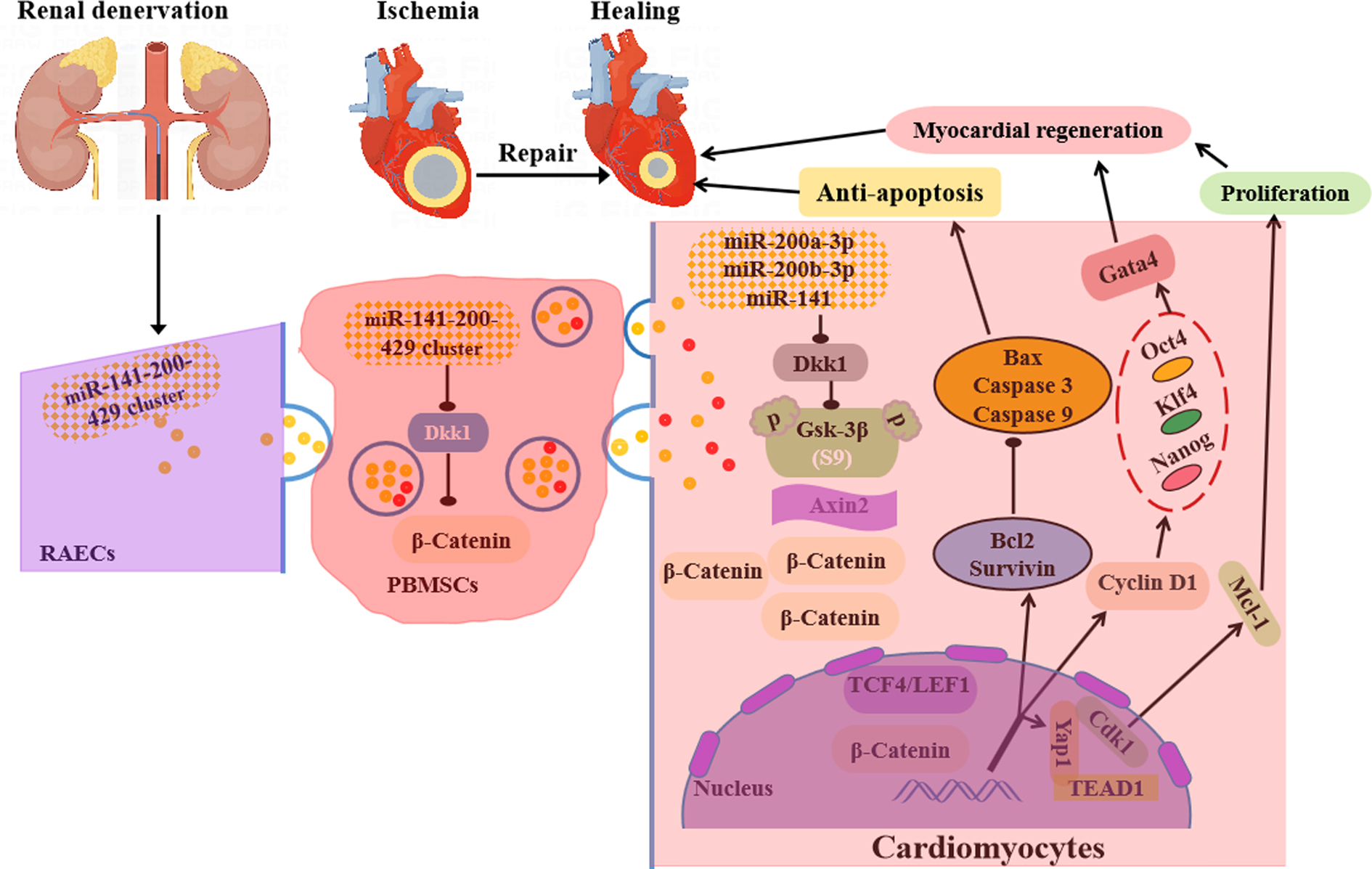

Supplement: Supplementary file 8 — Supporting Information [file CTM2-15-e70475-s001.jpg]
