# Supplementary material for: Peripheral blood mesenchymal stem cell‐derived exosomes improve renal sympathetic denervation efficacy through β‐catenin‐mediated cardiac reprogramming
Source: Clin Transl Med. 2025 Sep 5;15(9):e70475. doi: 10.1002/ctm2.70475 (PMC12411928; doi:10.1002/ctm2.70475)
Supplement: Supplementary file 9 — Supporting Information [file CTM2-15-e70475-s004.docx]

**Table S1 SeqTences of primers Tsed for gene amplification.**

| **Genes** | **Forward** | **Reverse** |
| --- | --- | --- |
| Acta1 | CCAGCCCTCCTTCATCG | TGGCGTACAGGTCCTTCCT |
| Bax | ATCGAGCAGGGCGAATG | TGTCCACGGCGGCAAT |
| β-catenin | TCTCATCAAGGCTACCG | CGAACCAGCAACTGAACTA |
| Bcl2 | CTGGGAGAACAGGGTACGATAA | GGCTGGGAGGAGAAGATGC |
| bFGF | AAGAGCGACCCTCACATCAAG | TTCGTTTCAGTGCCACATACC |
| Caspase 3 | ACCAGTTGAGGCAGAC | ACCCGAGTAAGAATGTG |
| Caspase 9 | CGAACTAACAGGCAAGCA | CCAAATCCTCCAGAACCA |
| c-Myc | TCCAGGACTGTATGTGGAGC | TCGTTGAGCGGGTAGGG |
| Cdk1 | CAATTAAACTGGCTGAT | ATATGGTGCCTATACTCC |
| Dkk1 | GGGGAAATTGAGGAAACC | CACAGTCTGATGATCGGAGA |
| GAPDH | GCATCCTGGGCTACACTG | TGGTCGTTGAGGGCAAT |
| Klf4 | GGAGGGAGACGGAGGAGTT | CTGAAGCCGACGAGGACA |
| Mcl-1 | GCATCGAACCGTTAGCAGA | GAAGAACTCCACAAACCCATC |
| miR-200a-3p | TAACACTGTCTGGTAACGATGT |  |
| miR-200b-3p | TAATACTGCCTGGTAATGATGAC |  |
| miR-200c-3p | TAATACTGCCGGGTAATGATGGA |  |
| miR-141 | TAACACTGTCTGGTAAAGATG |  |
| miR-429 | TAATACTGTCTGGTAATGCCGT |  |
| Nppa | CTTCCTCCTCGTTCTGGTG | TCTTCTAAAGGCATCTTGTCC |
| Oct4 | GTCGCCAGAAGGGCAAAC | CAGGGTGGTGAAGTGAGGG |
| P53 | TGACTGTACCACCATCCACTAC | TGTTCCGTCCCAGCAAG |
| Sox2 | CCCGTGGTTACCTCTTCTTCC | TACCGTTGATGGCCGTGCC |
| Suvivin | GACCACCGCATCTCCA | CAAATCGGGCTCGTTC |
| Yap1 | CCAGGAATGGCTTCAAGAT | AGAGCACAGACAGCGGACTA |
