# Supplementary material for: Peripheral blood mesenchymal stem cell‐derived exosomes improve renal sympathetic denervation efficacy through β‐catenin‐mediated cardiac reprogramming
Source: Clin Transl Med. 2025 Sep 5;15(9):e70475. doi: 10.1002/ctm2.70475 (PMC12411928; doi:10.1002/ctm2.70475)
Supplement: Supplementary file 10 — Supporting Information [file CTM2-15-e70475-s008.docx]

**Table S 2 The antibodies for WB, IHC, and IF**

| Name | Description | Company | Catalogue number | Applications |
| --- | --- | --- | --- | --- |
| α-Actin | alpha Actin | Bioss Inc. | Bs-0189R | WB, IF |
| α-SMA | Alpha-smooth muscle actin | GeneTex | GTX57619 | WB, IF |
| Acta1 | Alpha Actin skeletal muscle | [MyBioSource.com](https://www.biocompare.com/104355-MyBioSource-com/) | MBS8505782 | WB |
| Aurora B |  | [BosterBio](https://www.biocompare.com/104918-Boster-Biological-Technology/) | M00762-4 | WB, IF, IHC |
| Axin2 |  | [GeneTex](https://www.biocompare.com/100423-GeneTex/) | GTX105442 | WB, IHC |
| β-Catenin |  | Sigma-Aldrich | SAB4500541 | WB, IF, IHC |
| Bcl2 | B-cell lymphoma-2 | [GeneTex](https://www.biocompare.com/100423-GeneTex/) | GTX17715 | WB, IHC |
| Calnexin |  | [MyBioSource.com](https://www.biocompare.com/104355-MyBioSource-com/) | MBS2138476 | WB, IHC |
| Caspase 3 |  | [GeneTex](https://www.biocompare.com/100423-GeneTex/) | GTX636810 | WB, IHC |
| CD9 | Cluster of differentiation 9 | Proteintech | 20597-1-AP | WB, IF |
| CD31 | Cluster of differentiation 31 | [Bioss Inc.](https://www.biocompare.com/104473-Bioss-Inc/) | bs-0468R-PE | FCM |
| CD34 | Cluster of differentiation 34 | Becton Dickinson | 751621 | FCM |
| CD44 | Cluster of differentiation 44 | [Bioss Inc.](https://www.biocompare.com/104473-Bioss-Inc/) | bs-2507R-PE | FCM |
| CD45 | Cluster of differentiation 45 | [GeneTex](https://www.biocompare.com/100423-GeneTex/) | GTX44565 | FCM, IF |
| CD63 | Cluster of differentiation 63 | abcam | ab217345 | WB, IF |
| CD71 | Cluster of differentiation 71 | [MyBioSource.com](https://www.biocompare.com/104355-MyBioSource-com/) | MBS215085 | FCM |
| CD90 | Cluster of differentiation 90 | Becton Dickinson | 561973 | FCM |
| CD105 | Cluster of differentiation 105 | [Biorbyt](https://www.biocompare.com/104369-Biorbyt/) | orb488557 | FCM |
| CD133 | Cluster of differentiation 133 | [Biorbyt](https://www.biocompare.com/104369-Biorbyt/) | orb703845 | FCM |
| Cdk1 | Cyclin dependent kinase 1 | [NSJ Bioreagents](https://www.biocompare.com/106087-NSJ-Bioreagents/) | R30620 | WB |
| Cleaved caspase 3 |  | [R&D Systems](https://www.biocompare.com/100364-R-D-Systems/) | MAB835 | WB, IF |
| c-Myc |  | NSJ Bioreagents | RQ4860 | WB, IF |
| cTnI | Cardiac troponin I | [MyBioSource.com](https://www.biocompare.com/104355-MyBioSource-com/) | MBS8211896 | IF, IHC, WB |
| cTnT | Cardiac troponin T | [MyBioSource.com](https://www.biocompare.com/104355-MyBioSource-com/) | MBS423139 | IF, WB |
| Cyclin D1 |  | Sigma-Aldrich | SAB4502603 | WB |
| DAB2 | Disabled-2 | [MyBioSource.com](https://www.biocompare.com/104355-MyBioSource-com/) | MBS3015470 | IF, FCM |
| Dkk1 | Dickkopf-1 | [MyBioSource.com](https://www.biocompare.com/104355-MyBioSource-com/) | MBS850869 | WB, IF |
| GAPDH | Glyceraldehyde-3- phosphate dehydrogenase | [Novus Biologicals](http://www.biocompare.com/9776-Antibodies/119146-GAPDH-Antibody/?soids=1014,257&ppim=119146_1_1&ncatid=9776&dfp=true##) | NB300-221 | WB |
| Gata 4 | GATA binding protein 4 | [GeneTex](https://www.biocompare.com/100423-GeneTex/) | GTX113194 | WB, IF, IHC |
| GSK-3β | Glycogen synthase dinase 3 beta | [Affinity Biosciences](https://www.baidu.com/link?url=IBBCL93D0g_uc-P0b7f1tQrXfArFlPoXHOGJfbEwPKruussdP5spG4s78AVM-eHgTSe7ZHAEUTXBn5Ps7XxJ2WD0Nb6nyRRgtNh6H0oSx2e&wd=&eqid=b482c6b0000078e40000000366fd59b3) | DF7231 | WB, IF |
| Histone H3 |  | [GeneTex](https://www.biocompare.com/100423-GeneTex/) | GTX122148 | WB, IF, IHC |
| HSP70 | Heat shock protein 70 | [Biorbyt](https://www.biocompare.com/104369-Biorbyt/) | orb256610 | WB, IHC |
| Ki-67 |  | [MyBioSource.com](https://www.biocompare.com/104355-MyBioSource-com/) | MBS5306554 | WB, IF |
| Klf4 | Kruppel-like factor 4 | [GeneTex](https://www.biocompare.com/100423-GeneTex/) | GTX101509 | WB, IF, IHC |
| LEF1 | Lymphoid enhancer binding factor 1 | [GeneTex](https://www.biocompare.com/100423-GeneTex/) | GTX129186 | WB, IHC |
| MHC | Myosin Heavy Chain | [Miltenyi Biotec](https://www.biocompare.com/100196-Miltenyi-Biotec/) | 130-122-924 | IF, WB |
| Nanog |  | [GeneTex](https://www.biocompare.com/100423-GeneTex/) | GTX627421 | WB, IF, IHC |
| Oct4 | Octamer‑binding protein 4 | [GeneTex](https://www.biocompare.com/100423-GeneTex/) | GTX101497 | IF, IHC, WB |
| p53 |  | [GeneTex](https://www.biocompare.com/100423-GeneTex/) | GTX636395 | IF, IHC, WB |
| PH3 | Phospho-histone H3 | [Proteintech Group Inc](https://www.biocompare.com/102174-Proteintech-Group-Inc/) | 66863-1-Ig | IF, WB |
| p-GSK-3β S9 | Phosphorylated GSK-3β at ser 9 | Sigma-Aldrich | SAB4300287 | WB, IF |
| Runx1 | Runt related transcription factor-1 | [LifeSpan BioSciences](https://www.biocompare.com/100534-LifeSpan-BioSciences/) | LS-C353932 | IF, WB |
| SMAD2 | SMAD family member 2 | [GeneTex](https://www.biocompare.com/100423-GeneTex/) | GTX111075 | WB, IHC, IF |
| SOX2 | SRY-box 2 | [GeneTex](https://www.biocompare.com/100423-GeneTex/) | GTX35087 | WB, IHC |
| Survivin |  | [GeneTex](https://www.biocompare.com/100423-GeneTex/) | GTX100052 | WB, IF, IHC |
| TCF4 | Transcription factor 4 | Sigma-Aldrich | SAB1404449 | WB, IF, IHC |
| TCF7 | Transcription factor 4 | [GeneTex](https://www.biocompare.com/100423-GeneTex/) | GTX113851 | WB, IHC |
| TSG101 |  | [GeneTex](https://www.biocompare.com/100423-GeneTex/) | GTX70255 | WB, IF, IHC |
| Wnt2 | Wnt family member 2 | [MyBioSource.com](https://www.biocompare.com/104355-MyBioSource-com/) | MBS178216 | WB |
| YAP1 | Yes associated protein 1 | [GeneTex](https://www.biocompare.com/100423-GeneTex/) | GTX633541 | WB, IHC |
