# Supplementary material for: Peripheral blood mesenchymal stem cell‐derived exosomes improve renal sympathetic denervation efficacy through β‐catenin‐mediated cardiac reprogramming
Source: Clin Transl Med. 2025 Sep 5;15(9):e70475. doi: 10.1002/ctm2.70475 (PMC12411928; doi:10.1002/ctm2.70475)
Supplement: Supplementary file 11 — Supporting Information [file CTM2-15-e70475-s002.docx]

| Time | Baseline | 1w post-RD | 2w post-RD | 4w post-RD |
| --- | --- | --- | --- | --- |
| Heart rate (beats/min) | 82±8 | 88±5 | 83±6 | 82±6 |
| MABP ( mmHg) | 88±6 | 85±5 | 88±4 | 84±7 |
| LVEF (%) | 70.6±3.9 | 69.4±3.8 | 68.0±3.6 | 68.3±3.7 |
| Plasma BNP (pg/ml) | 89.8±9.2 | 97.0±3.0 | 90.6±11.6 | 96.3±18.4 |
| Plasma hs-cTnT (ng/l) | 0.045±0.007 | 0.054±0.009 | 0.054±0.006 | 0.052±19.7 |
| Plasma NE (ng/ml) | 0.44±0.08 | 0.60±0.11 | 0.54±0.07 | 0.50±0.007 |
| Plasma Ang-I (pM) | 10.22±2.47 | 11.53±2.21 | 11.22±1.35 | 10.45±2.28 |
| Plasma Ang-II (pM) | 91.01±20.64 | 92.78±17.07 | 91.23±16.26 | 92.13±19.94 |

**Table S3 LVEF, MABP, BNP, hs-cTnT, NE and Ang in NON-MI+RD pigs**
