# Supplementary material for: Peripheral blood mesenchymal stem cell‐derived exosomes improve renal sympathetic denervation efficacy through β‐catenin‐mediated cardiac reprogramming
Source: Clin Transl Med. 2025 Sep 5;15(9):e70475. doi: 10.1002/ctm2.70475 (PMC12411928; doi:10.1002/ctm2.70475)
Supplement: Supplementary file 12 — Supporting Information [file CTM2-15-e70475-s009.docx]

**Table S4 Sequences for miR-141-200-429 cluster mimics and β-catenin shRNAs.**

| **Names** | **Sense** | **Antisense** |
| --- | --- | --- |
| miR-141 mimics | UAACACUGUCUGGUAAAGAUGG | AUUGUGACAGACCAUUUCUACC |
| miR-200a-3p mimics | UAACACUGUCUGGUAACGAUGU | ACAUCGUUACCAGACAGUGUUA |
| miR-200b-3p mimics | UAAUACUGCCUGGUAAUGAUGA | UCAUCAUUACCAGGCAGUAUUA |
| NC mimics | UCACAACCUCCUAGAAAGAGUAGA | UCUACUCUUUCUAGGAGGUUGUGA |
| NC sponge | CCCAAGCTTACATaaATACCAGACAGTGTTATTCAAGAGA | CCGGAATTCTCAAGAGTAACACTGTCTG GTAttATGT |
| Ctnnb1 shRNAs | ACUAACAGCCGCUUUUCUGUCCAGAAAAGCGGCUGUUAGUCA |  |
| miR-141-miR-200a-  miR-200b-cluster sponges | acaggatccACATCGTTACCAGACAGTGTTAtatacACATCGTTACCAGACAGTGTTAacatcACATCGTTACCAGACAGTGTTAtcttcaACATCGTTACCAGACAGTGTTAacaggatccTCATCATTACCAGGCAGTATTAtatacTCATCATTACCAGGCAGTATTAacatcTCATCATTACCAGGCAGTATTAtcttcaTCATCATTACCAGGCAGTATTAacaggatccCCATCTTTACCAGACAGTGTTAtatacCCATCTTTACCAGACAGTGTTAacatcCCATCTTTACCAGACAGTGTTAtcttcaCCATCTTTACCAGACAGTGTTAttttttgaattcaca |  |
